# Discovery of novel Li SSE and anode coatings using interpretable machine learning and high-throughput multi-property screening

**DOI:** 10.1038/s41598-021-94275-5

**Published:** 2021-08-13

**Authors:** Shreyas J. Honrao, Xin Yang, Balachandran Radhakrishnan, Shigemasa Kuwata, Hideyuki Komatsu, Atsushi Ohma, Maarten Sierhuis, John W. Lawson

**Affiliations:** 1 KBR Wyle, Intelligent Systems Division, NASA Ames Research Center, Moffett Field, CA 94035 USA; 2 Research Division, Nissan North America, Santa Clara, CA 95051 USA; 3 Research Division, Nissan Motor Company, Yokosuka, Kanagawa, 237-8523 Japan; 4 Intelligent Systems Division, NASA Ames Research Center, Moffett Field, CA 94035 USA

**Keywords:** Batteries, Computational methods

## Abstract

All-solid-state batteries with Li metal anode can address the safety issues surrounding traditional Li-ion batteries as well as the demand for higher energy densities. However, the development of solid electrolytes and protective anode coatings possessing high ionic conductivity and good stability with Li metal has proven to be a challenge. Here, we present our informatics approach to explore the Li compound space for promising electrolytes and anode coatings using high-throughput multi-property screening and interpretable machine learning. To do this, we generate a database of battery-related materials properties by computing $\hbox {Li}^+$ migration barriers and stability windows for over 15,000 Li-containing compounds from Materials Project. We screen through the database for candidates with good thermodynamic and electrochemical stabilities, and low $\hbox {Li}^+$ migration barriers, identifying promising new candidates such as $\hbox {Li}_9\hbox {S}_3$N, $\hbox {LiAlB}_2\hbox {O}_5$, $\hbox {LiYO}_2$, $\hbox {LiSbF}_4$, and $\hbox {Sr}_4\hbox {Li}(\hbox {BN}_2)_3$, among others. We train machine learning models, using ensemble methods, to predict migration barriers and oxidation and reduction potentials of these compounds by engineering input features that ensure accuracy and interpretability. Using only a small number of features, our gradient boosting regression models achieve $\mathrm {R}^2$ values of 0.95 and 0.92 on the oxidation and reduction potential prediction tasks, respectively, and 0.86 on the migration barrier prediction task. Finally, we use Shapley additive explanations and permutation feature importance analyses to interpret our machine learning predictions and identify materials properties with the largest impact on predictions in our models. We show that our approach has the potential to enable rapid discovery and design of novel solid electrolytes and anode coatings.

## Introduction

High energy density batteries that are, both, safe from volatile reactions with air and water, and capable of quick charging, are the holy grail of the electric vehicles industry today^1–5^. While advances in controls and engineering of battery modules are paving the way for highly optimized batteries, theoretical limits on battery performance have been largely determined by the underlying choice of materials and electrochemistry^6,7^. Previous developments in battery materials were driven by long cycles of experiments involving several expensive and time-consuming synthesis-and-characterization loops. Recently, high-throughput computations of materials properties, made possible by the exponential growth in computational capabilities of supercomputers and cluster-computing facilities, have resulted in the development of large open-source databases like Materials Project^8^, OQMD^9^, AFLOWLIB^10^, etc. Such databases provide valuable opportunities for many data mining and machine learning (ML) approaches^11–18^. Over the past few years, materials science researchers have implemented ML models to predict stoichiometries and geometries^19–22^, atomization energies^23–26^, formation energies^27–29^, diffusion barriers^30^, band gaps^31,32^, and many other materials properties. When used in conjunction with experiments and computations, ML approaches allow for the rapid exploration and discovery of new materials at much lower cost, significantly speeding up the materials design process.

Traditional Li-ion batteries use flammable organic liquid electrolytes that are prone to fire-hazards. Furthermore, the conventional choice of carbon-based anodes limits the specific energies of these batteries to less than 300 Wh/kg^33^. Ionic liquid electrolytes have been widely studied as a safer alternative due to their non-volatility and thermal stability^34^, however their high viscosity, and thus relatively low conductivity, have limited their appeal^35^. An all-solid-state battery (ASSB) with a Li metal anode can address the safety issues as well as the demand for higher energy densities. The major obstacle to creating such a battery involves the development of solid electrolytes and protective anode coatings possessing high ionic conductivity, wide electrochemical window, good stability against Li metal, and inertness to environmental elements like water and air.

Computational approaches to understand ionic migration in solids include the nudged elastic band method (NEB)^36^ and molecular dynamics (MD) methods based on density functional theory (DFT)^37,38^, space topology analysis based on the Voronoi-Dirichlet partitioning of crystal space^39,40^, and bond valence (BV) estimation techniques^38,41–44^. Although DFT methods provide the most accurate measurement of migration barriers, they are extremely expensive, thus limiting their applicability for high-throughput screening approaches. The BV method, on the other hand, can be used to quickly identify low mismatch pathways between Li sites, representing probable $\mathrm {Li}^+$ transport paths in these structures. By linking the BV mismatch to the absolute energy scale using a Morse potential, percolating migration barriers can be extracted from the energy landscape in a quick and accurate manner^45^. While the accuracy of the BV approach is contingent upon the development of accurate empirical potentials, results show a consistent trend against DFT values^45,46^, making it a quick screening filter for identifying fast ion-conducting solid electrolytes and coatings.

A lot of recent work has focused on screening for fast Li-conductors. Recently, Xiao *et al.*^38^ used BV calculations to identify oxide candidates with low migration barriers from a set of 1000 Li-containing compounds in the inorganic crystal structure database (ICSD)^47^. In two other publications, He^48,49^, Zhang^50^, and others developed a platform for performing high-throughput screening for over 29,000 inorganic compounds from the ICSD using a combination of topology features, bond valence energies, and NEB calculations. Another study involved the use of topological analysis and ab-initio MD simulations to quantify key structural features of fast ion conductors^51^. First-principles calculations have also been performed to assess electrochemical stability for a wide range of electrolyte/electrode combinations^52,53^, where exceptional agreement with experimental results was found. Several studies have focused on screening for coatings for Li and Li-ion batteries^54–56^. In one particular study, it was found that nitrides have a significantly lower reduction potential than sulfides, oxides, and fluorides, making them more suitable for anode coatings^57^.

ML approaches to identify promising solid electrolytes are also getting more attention. Recently, Tian et al.^58^ performed clustering based on modified XRD patterns of the anion sub-lattice to identify 16 promising Li-conductors. Sendek *et al.*^59^ screened for Li superionic conductors using a logistic regression ML model trained on a relatively small set of 40 experimentally measured ionic conductivity values. The model identified 21 compounds as being possible superionic conductors, 8 of which were actually predicted to have an ionic conductivity $> 10^{-4}$ S/cm based on high temperature DFT-MD simulations^60^.

In the following sections, we describe our approach to quickly explore the Li compound space for promising solid electrolyte and anode coating candidates. First, we create an extensive database of battery-related materials properties of electrolyte candidates based on over 15,000 Li compounds catalogued by Materials Project^8^. For each compound, we compute both transport and stability based properties. Our database can be used for multi-property screening and is distinguished from previous work which has been primarily based on single property descriptors and smaller datasets. Second, we screen our database for new compounds and identify over 250 promising candidates spanning different chemistries and structures. We find many compounds with low 3D barriers and wide stability. These candidates include some well-known names as well as several novel and unexpected compounds, which have not been previously reported. Third, we train accurate and interpretable multi-property ML models to predict 3D migration barriers as well as oxidation and reduction potentials. The ML models will permit broader searches for materials outside the original database. Previously, only one group has reported a supervised ML model for solid electrolytes, based on single property predictions of ionic conductivity trained on a small experimental dataset^59^. Arguably, our ML model is an improvement over these previous results and is also the first to predict electrochemical stability. In addition, our models are completely interpretable, and allow us to explain individual machine learning predictions in a way no other models do. We believe our multi-property ML model will be a valuable tool for future electrolyte design.

## Database of Li compounds

To identify novel candidates for Li ASSB, we first create a database of 15,446 Li-containing compounds with their computed materials properties. Most well-known solid electrolytes have, in common, the same few characteristics - high ionic conductivity, wide electrochemical stability window, low electronic conductivity, good chemical, thermal, and mechanical stability etc. In order to identify good solid electrolytes and anode coatings, we focus on three important characteristics in this work: i. fast ionic migration; ii. wide electrochemical stability window; and iii. stability against Li metal.

### Ionic migration

Ionic conductivity is the property most often used to study ionic migration in solids. The ionic conductivity of a solid measures how easily an ion can move from one site to another through defects in the crystal lattice. While the ionic conductivity clearly depends on the underlying crystal structure, experimentally measured values are also greatly influenced by the synthesis method, sample preparation, and measurement technique^61^. Additionally, the availability of, and access to, experimental ionic conductivity data is mostly limited; the only way to obtain such data is by text mining through existing publications or having access to internal databases maintained by individual research groups. Even then, the total accessible data is quite small. At the same time, theoretical ionic conductivity measurements using DFT-MD simulations are also quite expensive and not ideal for a high-throughput based approach. Instead, we compute $\hbox {Li}^+$ migration barriers using the softBV tool^45^ developed by Chen *et al.* to measure ionic migration in Li compounds.

The softBV tool is based on the BV method, more generally used to examine the stability of chemical structures or estimate the oxidation state of atoms^42,62,63^. At the heart of the BV method is the valence sum rule which states that the sum of bond valences ($S_{ij}$) around any atom, *i*, with neighboring atoms *j*, should be equal to the atomic valence, ($V_i$) *i*.*e*., the oxidation state.

1 $$\begin{aligned} V_i\ =\ \sum _{j} S_{ij} \end{aligned}$$

$S_{ij}$, considered a measure of the electrostatic strength of a bond, can be written as a simple two-parameter algebraic equation:

2 $$\begin{aligned} S_{ij}=\left( \frac{R_0-R_{ij}}{b}\right) , \end{aligned}$$

where $R_{ij}$ is the observed bond length between atoms *i* and *j*, and $R_0$ and *b* are bond valence parameters that depend entirely on the nature of the bond. Every pair of elements can be fitted with unique $R_0$ and *b* values associated with their interaction. The BV mismatch is defined as

3 $$\begin{aligned} |\Delta V_i| = V_i-V^{id}_i, \end{aligned}$$

where $V^{id}_i$ is the ideal valence state of atom *i*. Sites in a crystal structure where $|\Delta V_i| \approx 0$ are considered accessible sites for atom *i*. The BV method postulates that paths between accessible sites along which $|\Delta V_i|$ remains sufficiently low represent probable ion transport pathways. Chen *et al.* linked the BV mismatch, $|\Delta V_i|$, to the energy scale, in order to compute activation energies, by developing a BV-based force field method using a general Morse-type interaction potential^45^. The isosurfaces of fixed $|\Delta V_i|$ then represent regions that atom *i* can reach with a certain activation energy.

According to the original definition of the valence sum rule, only interactions with atoms, *j*, in the first coordination shell are considered in Equation (1). However, it was later demonstrated that by incorporating information from higher coordination shells, $R_0$ and *b* can, in most cases, be refined further. The new parameters thus obtained by incorporating higher shell information are referred to as the bond softness parameters, and the resulting tool based on this approach is called softBV.

In addition to describing migration pathways between lattice sites, softBV can also find the lowest energy isosurfaces that percolate through the *X*, *Y*, and *Z* directions of the unit cell. The 1D barrier represents the lowest energy required by a diffusing species to hop across between two opposite faces of a unit cell, in any one of the three directions. The 2D and 3D barriers, similarly, represent the lowest energies required to hop between opposite faces in any two or all three directions, respectively. It should be evident that the 1D barrier $\le$ 2D barrier $\le$ 3D barrier for all solids. For the purpose of this study, we only use the 3D barriers. Supplementary Fig. S1 shows the 3D migration pathway for $\hbox {Li}^+$ in $\mathrm {Li}_3\mathrm {P}\mathrm {S}_4$, evaluated using the softBV tool. The lowest activation energy required to connect every point on the shaded pathway is the 3D migration barrier, and it provides a quantitative measure of the achievable Li-ion conductivity in $\mathrm {Li}_3\mathrm {P}\mathrm {S}_4$.

Using the softBV tool, we calculate 3D barriers for $\hbox {Li}^+$ diffusion in all 15,446 Li compounds in a high-throughput manner. While the accuracy of softBV depends on the accuracy of underlying bond softness parameters and empirical potential energy functions, a consistent trend between $\hbox {Li}^+$ barriers computed using softBV and DFT has been shown to exist^45,46^. It is important to remember that materials with potential for fast migration may not necessarily show high ionic conductivity unless there is a high concentration of mobile ions to carry the charge^64^. It is, thus, possible that Li compounds with extremely low 3D barriers have large associated defect formation energies that prevent them from being superionic conductors. The ionic conductivity of such materials can generally be enhanced by the introduction of additional charge carriers. Furthermore, since the BV approach does not allow for the structural relaxation of neighboring atoms that accompanies migration, softBV barriers tend to be overestimated in some cases^65^. Hence, softBV barriers should not be used to directly estimate the ionic conductivity.

### Electrochemical stability window

The electrochemical stability window of a material represents the electrode potential range in which it is neither oxidized nor reduced. We use the grand potential phase diagram approach^66,67^ to calculate the electrochemical stability window of materials. The grand potential phase diagrams represent phase equilibria that are open to Li. Here, the chemical potential of Li ($\mu _{Li}$) is the external input variable that can be controlled. The relevant thermodynamic potential to study phase equilibria is the Li grand potential, defined as

4 $$\begin{aligned} e\phi =E-\mu _{Li}N_{Li} \end{aligned}$$

where, *e* is the elementary charge, $\phi$ is the electrode potential, *E* is the total energy computed using DFT, and $N_{Li}$ is the number of Li atoms in the system. The resulting phase diagram provides information about the equilibrium phases at different values of $\mu _{Li}$.

As an example, we use the grand potential phase diagram of the ternary Li−P−S system to calculate voltage profiles depicting the lithiation and delithiation of $\mathrm {Li}_3\mathrm {P}\mathrm {S}_4$ (Supplementary Fig. S2). At low electrode potentials, we see that $\mathrm {Li}_3\mathrm {P}\mathrm {S}_4$ undergoes reduction and uptakes Li to form $\mathrm {Li}_2\mathrm {S}$ and P, while at higher potentials, $\mathrm {Li}_3\mathrm {P}\mathrm {S}_4$ is oxidized and loses Li forming $\mathrm {Li}\mathrm {S}_4$ and $\mathrm {P}_2\mathrm {S}_7$. The electrochemical stability window of $\mathrm {Li}_3\mathrm {P}\mathrm {S}_4$ ($1.7-2.2$ V) is the range in which no lithiation or delithiation occurs *i.e.* where Li uptake is zero.

The grand potential analyses performed in this study include only the lowest energy phase at each composition. Thus, only 8,924 Li compounds in the database have reported electrochemical stability windows. In the case of meta-stable compounds that do not lie on the convex hull, the stability window is represented by the oxidation and reduction potentials of their decomposition components in the grand potential phase diagram. We use the DFT energies obtained from Materials Project for our calculations.

### Stability against Li metal

Stability against Li metal represents a material’s inertness to lithium. A material is considered stable if it does not undergo spontaneous reaction with Li at 0 V. Thus, materials that are stable against Li have a reduction potential (*vs.* Li/Li+) of 0 V. According to Supplementary Fig. S2, $\mathrm {Li}_3\mathrm {P}$ and $\mathrm {Li}_2\mathrm {S}$ are the only two phases in the Li−P−S system that are stable against Li metal.

Besides the three characteristics described above, we include in our database DFT computed values of energy above the convex hull ($E_{hull}$) and band gap ($E_{g}$) for all 15,446 Li compounds, also obtained from Materials Project. $E_{hull}$, which is a measure of thermodynamic stability at 0 K, is defined as the vertical distance of a phase from the convex hull in terms of energy per atom. A stable compound has $E_{hull}= 0\,\hbox {eV}$. Compounds with non-zero $E_{hull}$ values are thermodynamically unstable at 0 K, although they may be stabilized at higher temperatures through entropic contributions. We use $E_{g}$ as a measure of the electronic conductivity in a solid. Materials with $E_{g} > 0\,\hbox {eV}$ are known to be bad electronic conductors. In the future, we plan to extend the database to include other properties like elastic moduli, stability in water and air, etc.

## Screening

We use the database generated in section "Database of Li compounds" to screen for promising solid electrolyte and anode coating candidates. To identify fast Li-conducting solid electrolytes, we focus on materials with low migration barriers. We select an arbitrary cutoff of 0.5 eV, and screen for all Li compounds with 3D barriers $\le 0.5 \,\hbox {eV}$.

For protective coatings on the anode, stability against Li metal on one side, and the electrolyte on the other, is more important. Hence, we identify compounds with reduction potentials (*vs.* Li/Li+) of 0 V and an electrochemical stability window wider than 1 V. We restrict the 3D barriers of coatings to 1 eV, to achieve a good balance between electrochemical stability and ionic conduction. Because coatings are generally applied as thin-films, they allow for a comparatively low ionic conductivity.

To ensure that our screened solid electrolytes and coatings are also thermodynamically stable and possess low electronic conductivities, we add a second filtering step to only include compounds with $E_{hull} \le 30 \,\hbox {meV}$ and $E_{g} \ge 1 \,\hbox {eV}$. We use a relatively safe lower bound of 1 eV to ensure minimal electronic conduction across the solid electrolyte or coating layer, accounting for the fact that local and semi-local DFT functionals tend to underestimate band gaps^68^. Finally, compounds with transition metal atoms are generally known to be susceptible to reactions with Li, as transition metals have many stable oxidation states. We, therefore, filter out most compounds with d- and f-block elements.

Although we calculate electrochemical stability windows only for the lowest energy phase at every composition, limiting our search to candidates with $E_{hull} \le 30 \,\hbox {meV}$ ensures that the stabilities of all identified compounds at a given composition are fairly similar. It should be noted that since the grand potential phase diagram depends on all existing phases in a given chemical subspace, it is prone to change when new phases are discovered or get added to Materials Project. This would affect the electrochemical stability windows of all compounds within the subspace. However, many of these systems are fairly well-studied and this should not be a major issue.

**Figure 1:**
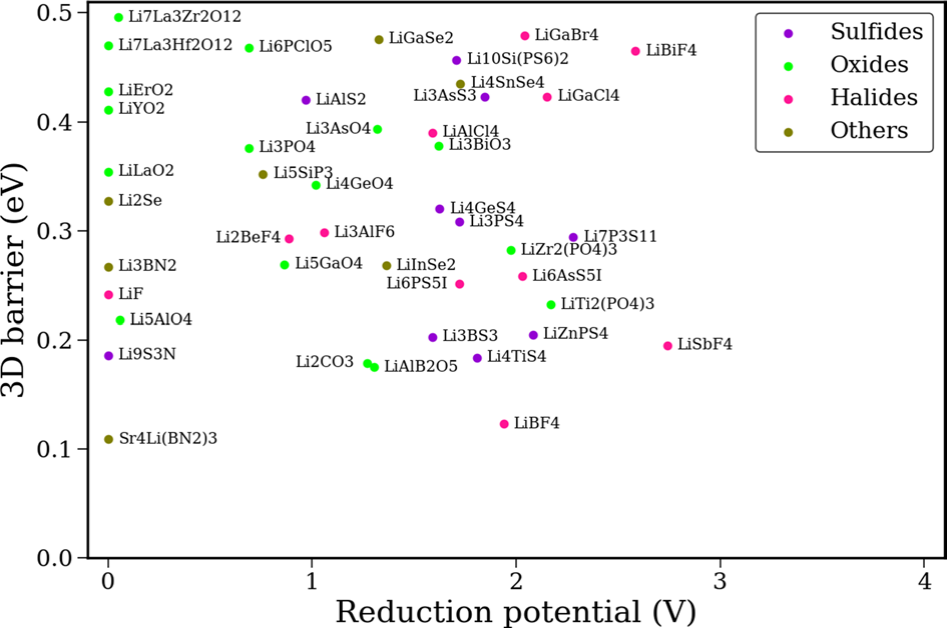
Promising electrolyte and anode coating candidates identified through the screening approach. Complete screening results can be found in Supplementary Figs. S3–S6 and Supplementary Table S1. [3D barrier $\le 0.5 \,\hbox {eV}$, $E_{hull}\le 30 \,\hbox {meV}$, $E_g\ge 1\,\hbox {eV}$].

We identify over 250 promising solid electrolyte candidates using the above criteria. Few of them are highlighted in Fig. 1. The screened compounds are classified based on their chemistries into sulfides, halides, oxides, and other compounds. Supplementary Figs. S3–S6 show expanded results for each class. Among the sulfide compounds, well-known superionic conductors like $\hbox {Li}_7\hbox {P}_3\hbox {S}_{{11}}$ and $\hbox {Li}_3\hbox {PS}_4$ are identified^69,70^. While $\hbox {Li}_{{10}}\hbox {GeP}_2\hbox {S}_{{12}}$ (LGPS) does not show up due to its marginally higher $E_{hull}$ (32 meV), its Si counterpart ($\hbox {Li}_{{10}}\hbox {SiP}_2\hbox {S}_{{12}}$) gets picked up by our screening algorithm^71^. In addition, we identify several new sulfides, including $\hbox {Li}_9\hbox {S}_3\hbox {N}$, as promising electrolyte candidates based on their low 3D barriers. Among halides, the argyrodite $\hbox {Li}_6\hbox {PS}_5$I is identified^72^, but $\hbox {Li}_6\hbox {PS}_5$Br and $\hbox {Li}_6\hbox {PS}_5$Cl are excluded due to their higher $E_{hull}$ values. Other known fast Li-conductors like $\hbox {Li}_3\hbox {YBr}_6$ (0.55 eV), $\hbox {Li}_3\hbox {InCl}_6$ (0.59 eV), $\hbox {Li}_3\hbox {ScCl}_6$ (0.56 eV) fall just above the 3D barrier cutoff of 0.5 eV and are also excluded. However, both $\hbox {LiBF}_4$ and $\hbox {LiSbF}_4$ are identified as having extremely low 3D barriers. $\hbox {LiAlB}_2\hbox {O}_5$ is the best performing oxide identified through screening, having a low 3D barrier of 0.175 eV. Other screened oxides include NASICON-type solid electrolytes like $\hbox {LiTi}_2(\hbox {PO}_4)_3$ and $\hbox {LiZr}_2(\hbox {PO}_4)_3$, as well as garnets like $\hbox {Li}_7\hbox {La}_3\hbox {Zr}_2\hbox {O}_{{12}}$ (LLZO) and $\hbox {Li}_7\hbox {La}_3\hbox {Hf}_2\hbox {O}_{{12}}$ (LLHfO), all of which have measured ionic conductivities ranging between $10^{-5}$ to $10^{-3}$ S/cm^2^. $\hbox {Li}_2\hbox {CO}_3$ is also identified as a fast Li-conductor with a low 3D migration barrier. DFT studies have confirmed the low migration barrier for $\hbox {Li}_2\hbox {CO}_3$^73^, however it has been shown that an increase in its charge carrier concentration through the introduction of intrinsic defects or dopants is required to overcome the relatively high defect formation energies in this compound^74^.

For anode coatings, the stringent requirement of stability against Li metal leaves us with only 26 compounds, including some binaries like $\hbox {Li}_2\hbox {O}$, $\hbox {Li}_2\hbox {S}$, $\hbox {Li}_2\hbox {Se}$, and Li*X* ($X=$ halide) which have already been tested as thin-film coatings^75,76^ (see Supplementary Table S1). We identify $\hbox {Sr}_4\hbox {Li}(\hbox {BN}_2)_3$ as an extremely promising anode coating candidate with a low 3D barrier and wide electrochemical stability window. Similarly, $\hbox {LiYO}_2$ and $\hbox {Li}_7\hbox {La}_3\hbox {Hf}_2\hbox {O}_{{12}}$, both of which have been previously studied as potential solid electrolyte candidates^77,78^, also get picked up by the screening algorithm. The success of such thin-film coatings largely depends on striking the right balance between thickness of the coating, achieved by deposition or chemical reaction methods, and complete coverage of the Li metal anode surface.

$\hbox {Li}^+$ migration pathways for a few chosen electrolyte and anode coating candidates are shown in Supplementary Fig. S7.

## Machine learning

Screening, as described above, helps us explore the database of Li compounds for promising solid electrolyte and anode coating candidates. ML, on the other hand, provides us a way to not only extend this search to materials outside the database, but to also explain individual predictions and generate model-level insights that can be used for designing new compounds. Training ML models on properties within the database gives us the ability to quickly test new Li compounds in the future without the need for explicit computations. Additionally, ML models can solve the problem of estimating 3D barriers for compounds for which softBV parameters have not yet been calculated.

We train independent ML models using Li compounds in the database to predict 3D barriers and electrochemical stability windows. ML regression models take a vector $x\in {{\mathrm{I\!R}}}^n$ as input and return a value *y*. To utilize these models for predictions, we therefore construct a vector based data representation that encodes relevant physical information about the crystal structure and/or composition of the compounds. We compare the performance of two ensemble learning algorithms on our regression tasks – random forests (RF)^79^ and gradient boosting decision trees (GB)^80,81^. Ensemble methods combine predictions from several base estimators to reduce bias and variance, thus improving performance. They work extremely well with small and medium sized datasets that have a mix of categorical and continuous features spanning various scales. As an additional bonus, ensemble algorithms do not require feature scaling of components of input vectors. We use the implementations of RF and GB regression available from the sci-kit learn python package^82^. We perform hyperparamter selection using a grid-based search of the hyperparameter space and ten-fold cross-validation. $R^2$ values are obtained by averaging over 20 randomized 90%-10% training-test set splits and are reported for the held-out test set only.

### 3D barriers

For the task of predicting 3D barriers, we use the crystal structures of Li compounds as input. We identify features that are both physically motivated and easy to compute in order to represent the crystal structures. Based on previous research^2^, we know that structures with a large number of connected sites available for mobile ions to occupy, and small migration barriers between these sites, are ideal for fast ionic migration. To leverage this information, we include features describing the Li concentration, local geometry, sublattice chemistry, and topology into our ML models. We use several existing features, like those developed by Sendek *et al.*^59^ and Ward *et al.*^83^, as well as introduce some new ones. We perform feature selection to identify a final set of 22 features that provide a good balance between accuracy and simplicity in our model. The complete list is shown in Table 1 and described in Supplementary section S1.

**Table 1:** List of 22 structure-based features used to predict 3D barriers for $\hbox {Li}^+$ migration in Li compounds. A complete description of the individual features can be found in Supplementary section S1.

No.	Feature	Acronym
1.	Li atomic fraction	Li
2.	Mean Li neighbor count^59^	LNC
3.	Mean Li–Li bonds per Li^59^	LLB
4.	Mean sublattice neighbor count^59^	SNC
5.	Mean sublattice bond ionicity^59^	SBI
6.	Mean electronegativity of sublattice^59^	ENS
7.	Mean Li–Li separation distance^59^	LLSD
8.	Mean Li-anion separation distance^59^	LASD
9.	Mean anion-anion separation distance^59^	AASD
10.	Mean neighbor distance variation^83^	NDV
11.	Mean ordering parameter shell 1^83^	OP_1
12.	Mean ordering parameter shell 2^83^	OP_2
13.	Mean ordering parameter shell 3^83^	OP_3
14.	Diameter of largest free sphere^84^	DLFS
15.	Mean Straight Line Path Width^59^	SLPW
16.	Sublattice packing fraction^59^	SPF
17.	Max packing efficiency^83^	MPE
18.	XRD principal component 1	XRD_1
19.	XRD principal component 2	XRD_2
20.	XRD principal component 3	XRD_3
21.	XRD principal component 4	XRD_4
22.	XRD principal component 5	XRD_5

We use features like *Li atomic fraction* (Li), *mean Li neighbor count* (LNC), and *mean Li-Li bonds per Li atom* (LLB) to feed information about the concentration and arrangement of Li atoms to our models. Other features like *mean sublattice neighbor count* (SNC), *mean sublattice bond ionicity* (SBI), and *mean electronegativity of the sublattice* (ENS) provide similar details about the sublattice elements *i.e.* non-Li constituents in the compounds. Local geometry is also captured in our models through features like *mean Li-Li, Li-Anion*, and *Anion-Anion separation distances* (LLSD, LASD, AASD), *mean neighbor distance variation* (NDV), and *mean ordering parameters* (OP_1, OP_2, OP_3).

Topological features provide details about the channels available for $\hbox {Li}^+$ diffusion within the crystal structure. Such information is incorporated into the model through features including *diameter of the largest free sphere* (DLFS), calculated using the Zeo++ tool^84^, *mean straight line path width* (SLPW), *sublattice packing fraction* (SPF), and *maximum packing efficiency* (MPE).

Additionally, in order to also include a single representative fingerprint of the entire crystal structure, we use a reduced version of the powder X-ray diffraction (XRD) pattern, consisting of the first five eigenvectors (XRD_1 to XRD_5) calculated using principal component analysis, as additional inputs to our models. The fraction of variance explained by the first five principal components together is 0.65. Our final choice of 22 assorted features are meant to provide high accuracy in our models while still maintaining interpretability.

### Oxidation and reduction potentials

Since electrochemical stability windows are calculated for a single (lowest energy) phase at each composition, we decide to use only the composition of Li compounds as input for training the corresponding ML models. We break the stability window prediction problem into two tasks: predicting oxidation potentials (*vs.* Li/Li+) and reduction potentials (*vs.* Li/Li+), using the same set of input features for both tasks.

As before, we test an assortment of different features to represent the composition of Li compounds in the database. These are based on physical and chemical properties of individual elements present in the compound, weighted by composition. They include eight simple element properties, each measured through three statistics – mean, maximum, and range – resulting in a total of 24 distinct features. In addition, we introduce 4 new features based on oxidation states. The first two measure the total difference between the oxidation states of elements present in the compound and their lowest, or highest, possible oxidation states. For compound *X*, the two features are given by

$$\begin{aligned} {oxid. state - min. oxid. state (X)}= & {} \sum \limits _{i \in \{X\}} |\text {V}_i^X - min~{\{\text {V}}_i\} |\\ {max. oxid. state - oxid. state (X)}= & {} \sum \limits _{i \in \{X\}} |max~{\{\text{V}}_i\} - \text{V}_i^X| \end{aligned}$$

where, $\{X\}$ is the set of elements present in *X*, $V_i^X$ is the oxidation state of element *i* in compound *X*, and $\{V_i\}$ is the set of possible oxidation states of *i*. The remaining two features measure the same quantity, but are weighted by the Pauling electronegativities of the elements.

$$\begin{aligned} {(oxid. state - min. oxid. state) * E\_neg~(X)}= & {} \sum \limits _{i \in \{X\}} |\text {V}_i^X - min~{\{\text{V}}_i\} | * \text{EN}_i\\ {(max. oxid. state - oxid. state) * E\_neg~(X)}= & {} \sum \limits _{i \in \{X\}} |max~{\{\text{V}}_i\} - \text{V}_i^X| * \text {EN}_i \end{aligned}$$

The final list of 28 features is given in Table 2.

**Table 2:** List of 28 element property features used for predicting oxidation and reduction potentials (*vs.* Li/Li+).

Composition-weighted element properties	Statistics
**Standard features**	
Atomic number	Mean
Electronegativity	Maximum
Valence d-electrons	Range
Unfilled d-electrons	
Unfilled p-electrons	
Band gap	
Magnetic moment	
Melting temperature	
**New oxidation state features**	
oxid. state − min. oxid. state	
max. oxid. state − oxid. state	
(oxid. state − min. oxid. state) * E_neg	
(max. oxid. state − oxid. state) * E_neg	

Each of eight standard element properties are weighted by composition and measured through three different statistics to obtain the first 24 features. The other 4 are new oxidation state features introduced in “Oxidation and reduction potentials”.

### Shapley explanations and permutation feature importances

To interpret a ML model, it is useful to know which features have the highest impact on predictions. SHapley Additive exPlanations (SHAP)^85^ and Permutation Feature Importance analysis (PFI)^79^ are two model inspection techniques that we use to explain the output from our ML models. Shapley explanations use a game theoretic approach to break down individual predictions. Specifically, SHAP measures the impact of having a certain value for a given feature in comparison to the feature taking some baseline value.

On the other hand, PFI provides an overall metric of the most important features for a given model. It measures the change in model score when a single feature vector is randomly permuted; a higher score reflects a higher dependence of the model on the corresponding feature. The shap and rfpimp^86^ python packages are used to calculate SHAP and PFI values in this work. We report SHAP values for both training and test data, whereas PFI is measured, using $R^2$ values, on the test set only.

Although tree-based methods also provide an inbuilt measure of feature importance, based on the decrease in impurity, they are always computed on the training set and, therefore, do not reflect the predictive ability of the feature on unseen data. Furthermore, impurity-based feature importance schemes are strongly biased towards high cardinality features. PFI does not exhibit any such biases. It must be noted that PFI, just like impurity-based metrics, can still be affected by the presence of highly correlated features. When a correlated feature is permuted, the model still has access to the feature through its correlated counterpart, thereby lowering the apparent importance value for both features. A common solution, and one we use for the oxidation and reduction potentials models, is to cluster the correlated features prior to performing feature analysis.

## Results and discussion

### 3D barriers

The structure-based features introduced above are used to train RF and GB regression models to predict 3D barriers. To avoid outliers, we restrict our models to compounds with 3D barriers $\le$ 5 eV. Table 3 shows the cross-validated results on the barrier prediction task. We see that the GB model performs slightly better than the RF model, providing $\textit{R}^2$ values of 0.86 on average. To study the importance of feature selection, we also compare the performance of our input features against other structure-based representation schemes from literature. We find that our set of 22 input features performs better than all others at the 3D barrier prediction task. Supplementary Fig. S8 shows parity plots comparing the 3D barriers predicted using the GB model against barriers computed using softBV.

**Table 3:** The cross-validated performance of our RF and GB models trained using 22 input features on the 3D barrier prediction task.

Features + model	$R^2$	Vector length
Current work + GB	0.86	22
Current work + RF	0.84	22
Sendek conductivity features^59^ + GB	0.76	20
Coulomb matrix^23^ + GB	0.72	200
Powder X-ray diffraction pattern + GB	0.70	128

Also compared are three other structure-based representations from literature (with the same GB model in each case).

Although our database contains 1D, 2D, and 3D migration barriers calculated by softBV, we only use the 3D barriers for screening and ML. In isotropic materials, where ionic conduction is equally fast in all three dimensions, the three barriers are equal. Other compounds like $\hbox {LiFePO}_4$, $\hbox {LiCoO}_2$ etc. have dominant 1D or 2D conduction pathways^87,88^, and thus have 1D and 2D barriers that are significantly lower than their 3D barrier. However, crystal defects and imperfections can potentially obstruct these low barrier conduction pathways, and special material preparation and processing is needed to take advantage of them. The 3D barrier provides a much better estimate of the overall ionic migration in these compounds.

**Table 4:** Li compounds identified as possible superionic conductors by the Sendek model^59^ are arranged in increasing order of their softBV 3D barriers.

No.	mp_id	Formula	3D barrier (eV)	DFT-MD predicted ionic conductivity $> 10^{-4}\,\hbox {S/cm}$
1.	mp-558219	SrLi($\hbox {BS}_2)_3$	0.340	No
2.	mp-532413	$\hbox {Li}_5\hbox {B}_7\hbox {S}_{{13}}$	0.487	Yes
3.	mp-643069	$\hbox {Li}_2\hbox {HIO}$	0.519	Yes
4.	mp-7744	$\hbox {LiSO}_3\hbox {F}$	0.529	Yes
5.	mp-569782	$\hbox {Sr}_2\hbox {LiCBr}_3\hbox {N}_2$	0.548	Marginal
6.	mp-676109	$\hbox {Li}_3\hbox {InCl}_6$	0.595	Yes
7.	mp-676361	$\hbox {LiErCl}_6$	0.664	Yes
8.	mp-22905	LiCl	0.735	No
9.	mp-559238	$\hbox {CsLi}_2\hbox {BS}_3$	0.780	Yes
10.	mp-29410	$\hbox {Li}_2\hbox {B}_2\hbox {S}_5$	0.972$^{d}$	Yes
11.	mp-34477	$\hbox {LiSmS}_2$	0.977	No
12.	mp-8430	KLiS	1.720$^{d}$	No
13.	mp-8751	RbLiS	1.727$^{d}$	No
14.	mp-554076	$\hbox {BaLiBS}_3$	2.035	No
15.	mp-866665	$\hbox {LiMgB}_3$($\hbox {H}_9$N)$_2$	2.330$^{d}$	Yes
16.	mp-19896	$\hbox {Li}_2\hbox {GePbS}_4$	2.835	No
17.	mp-561095	$\hbox {LiHo}_3\hbox {Ge}_2$($\hbox {O}_4$F)$_2$	3.259	No
18.	mp-15791	$\hbox {LiErS}_2$	4.698$^{d}$	No
19.	mp-15790	$\hbox {LiHoS}_2$	4.749$^{d}$	No
20.	mp-15789	$\hbox {LiDyS}_2$	4.872	No
21.	mp-15797	$\hbox {LiErSe}_2$	4.884	Marginal

Where calculated barriers are missing, ML predictions are used instead. The rightmost column indicates whether the compound was predicted to have an ionic conductivity > 10$^{-4}$ S/cm based on high temperature DFT-MD simulations^60^.

$$^{d}$$ ML prediction

To test the validity of this idea, we consider the 21 compounds identified as being possible superionic conductors by the Sendek model^59^. Table 4 ranks the compounds in increasing order of their softBV 3D barriers. Where the calculated barriers are missing, we use predictions from our GB model instead. Among the 21 compounds, 8 are predicted to have an ionic conductivity > 10$^{-4}$ S/cm based on high temperature DFT-MD simulations, while 2 more are identified as marginal cases^60^. 8 of these 10 total compounds also have a low calculated 3D barrier and occupy the top half of the table. However, our models estimate much larger barriers for $\hbox {LiMgB}_3(\hbox {H}_9\hbox {N})_2$ as well as for $\hbox {LiErSe}_2$, which is structurally similar to $\hbox {LiErS}_2$. On the other hand, our models also perform well on the remaining 11 compounds that the Sendek model identifies as being superionic, but for which DFT-MD simulations predict ionic conductivities $< 10^{-4}$ S/cm. We estimate high 3D barriers for all but one of these compounds. The exception, $\hbox {SrLi}(\hbox {BS}_2)_3$, has the lowest calculated 3D barrier among the 21 compounds and is explored further below. LiCl, which appears to have a moderately low 3D barrier, is already identified as a promising anode coating candidate in "Screening" section. It must be noted that the LiCl structure identified by us is the DFT ground state structure, and slightly differs from the one identified by the Sendek model. Overall, our calculated and predicted 3D barriers show good agreement with high temperature DFT-MD simulation results.

**Figure 2:**
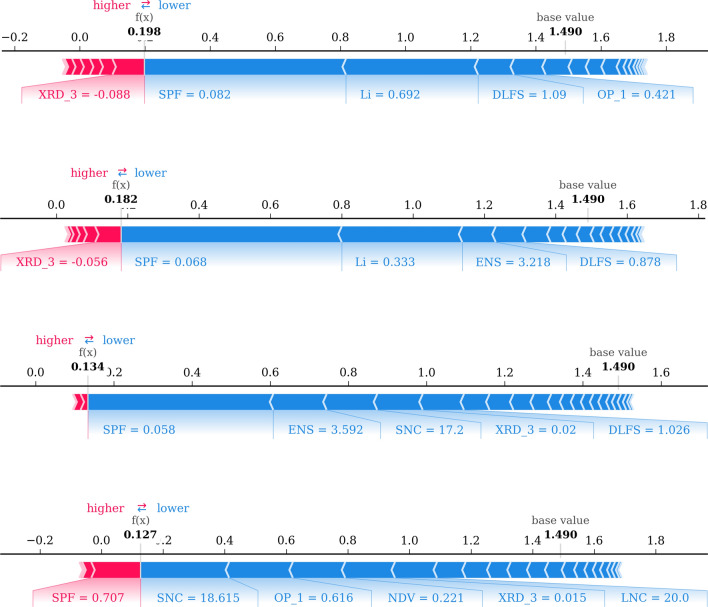
Shapley explanations for individual predictions made by the GB model trained on 3D barriers. Red arrows represent feature effects that drive the predicted 3D barrier higher, while blue arrows represent those that drive the prediction lower. The lengths of the arrows indicate the magnitudes of the effects. From top to bottom: $\hbox {Li}_9\hbox {S}_3\hbox {N}$, $\hbox {Li}_2\hbox {CO}_3$, $\hbox {LiBF}_4$, and $\hbox {Sr}_4\hbox {Li}(\hbox {BN}_2)_3$.

In addition to being good at predicting $\hbox {Li}^+$ migration barriers, our models are also interpretable. Fig. 2 shows Shapley explanations^89^ for individual predictions made by our GB model. Red arrows represent feature effects that drive the predicted 3D barrier higher, while blue arrows represent those that drive the prediction lower. The lengths of the arrows indicate the magnitude of the effects, referred to as SHAP values. We first consider 3D barrier predictions of compounds identified by the screening model as potential solid electrolyte candidates in "Screening" section. These are training set predictions that give us an insight into how the GB model uses input features to make decisions. We see that $\hbox {Li}_9\hbox {S}_3\hbox {N}$, the most promising sulfide compound, has an extremely small ${\textit{sublattice packing fraction}}~\hbox {(SPF)} = 0.082$ and extremely large $\textit{Li fraction} ~\hbox {(Li)} = 0.692$, driving its 3D barrier prediction from the base value of 1.490 eV, which is the GB model’s average prediction over the entire training set, down to 0.198 eV. Similarly, $\hbox {Li}_2\hbox {CO}_3$ also has a very small $\hbox {SPF} = 0.068$, moderate ${\textit{Li fraction}} = 0.333$, as well as high ${\textit{mean electronegativity of sublattice}}~(ENS) = 3.218$, resulting in a similarly low 3D barrier prediction of 0.182 eV. $\hbox {LiBF}_4$, on the other hand, does not have a large *Li fraction*. However, a small $\hbox {SPF} = 0.058$, high $\hbox {ENS} = 3.592$, and lower-than-average *sublattice neighbor count* (SNC) = 17.20, all drive its 3D barrier prediction down to 0.134 eV. All three compounds have reasonably large *diameters of largest free spheres* (DLFS). $\hbox {Sr}_4\hbox {Li}(\hbox {BN}_2)_3$, with one of the lowest computed 3D barriers, is a peculiar case. A small ${\textit{Li fraction}} = 0.071$ and extremely large $\hbox {SPF} = 0.707$, which in fact drives the prediction higher for $\hbox {Sr}_4\hbox {Li}(\hbox {BN}_2)_3$, are counterbalanced by a low $\hbox {SNC} = 18.615$ and several other negative contributions from local geometry based features.

We use similar plots to explain GB predictions for compounds from Table 4 with contradicting softBV and high temperature DFT-MD results. We see that $\hbox {LiErSe}_2$ has an extremely large predicted 3D barrier of 4.889 eV, even though its ionic conductivity is predicted to be marginal by DFT-MD. Supplementary Fig. S9 reveals that the the major contributors driving the 3D barrier prediction of $\hbox {LiErSe}_2$ higher are a large SPF = 0.384, high *maximum packing efficiency* MPE = 0.522, and low ENS = 2.113, all indicators of sluggish $\hbox {Li}^+$ migration. Similarly, $\hbox {LiMgB}_3(\hbox {H}_9\hbox {N})_2$ is also predicted to have an ionic conductivity $> 10^{-4}\,\hbox {S/cm}$ by DFT-MD, but its small $\hbox {SPF} = 0.126$ is more than made up for by a very high $\hbox {SNC} = 34.5$, extremely small $\textit{Li fraction} = 0.04$ , and low ${\textit{mean sublattice bond ionicity}} ~\hbox {(SBI)} = 0.284$ and $\hbox {ENS} = 2.213$, resulting in a high predicted 3D barrier of 2.330 eV. Finally, although $\hbox {SrLi}(\hbox {BS}_2)_3$ has a small ${\textit{Li fraction}} = 0.09$ and above-average $\hbox {SPF} = 0.279$, atypical of fast $\hbox {Li}^+$ conductors, it also has extremely low $\hbox {SNC} = 10.30$ and $\hbox {LNC} = 11.0$, and large $\hbox {DLFS} = 1.111$, explaining its low barrier prediction.

**Figure 3:**
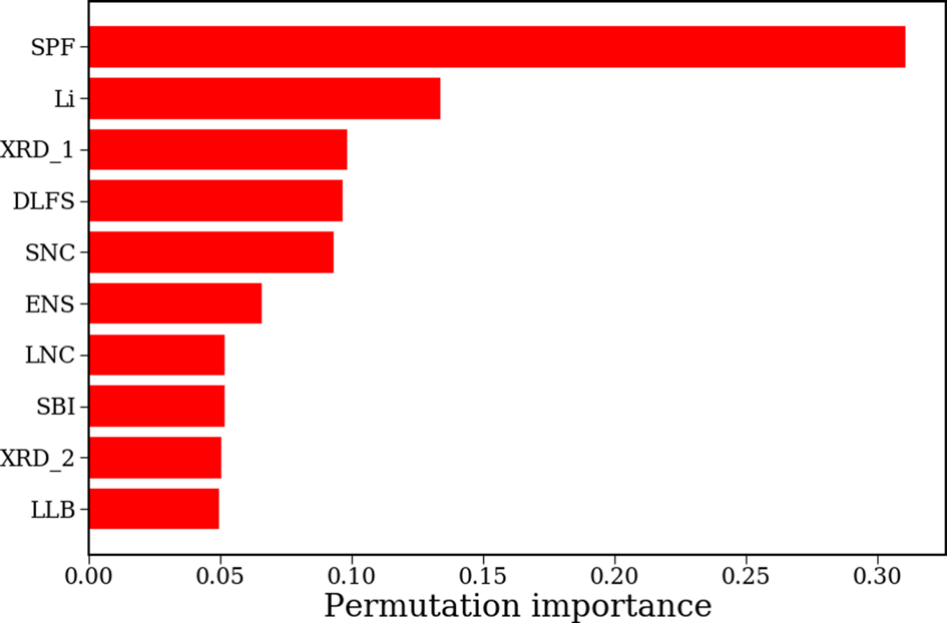
Permutation feature importance plot for the 3D barrier GB model showing the decrease in $R^2$ values upon random permutation of individual feature vectors. Higher values indicate a larger impact on predictions. Only features with the top 10 PFI scores are shown.

In addition to providing explanations for individual predictions, our ML models can also be used to study the impact any given feature has on overall predictions through PFI analysis. The PFI values measured on the test set of the GB model are shown in Fig. 3. It is evident from looking at the figure that SPF, by far, has the largest impact on overall predictions of 3D barriers. The feature importance plot indicates that if SPF values were to be randomly permuted for the test set, the resulting $R^2$ of the GB model would drop by $\sim 0.3$. In addition to SPF, we see other features discussed above like Li, SNC, and DLFS also have high impact on overall predictions. Both, SPF and DLFS, measure the void space inside the crystal structure available for $\hbox {Li}^+$ ions to diffuse through, making them excellent predictors of ionic migration. A low SPF, or wide DLFS, implies a low 3D barrier and, thus, fast ionic migration. Similarly, a high *Li fraction* or low SNC indicates a higher proportion of candidate Li sites for $\hbox {Li}^+$ to hop onto, also favoring fast migration. Although XRD_1, as a feature, is hard to interpret, its overall importance highlights the fact that crystal geometry largely determines the $\hbox {Li}^+$ migration barrier in these compounds.

Supplementary Fig. S10 shows a heatmap of the Pearson correlation coefficients between each pair of input features and between individual features and the 3D barrier. These coefficients measure the strength of the linear association between two variables, taking values between -1 and 1. Positive values imply a positive correlation whereas negative values imply an inverse correlation. We observe the same positive and negative correlations discussed in the paragraphs above, between the top five features and the 3D barrier.

### Oxidation and reduction potentials

Just like the 3D barriers, we also train ML models to predict oxidation and reduction potentials. Table 5 provides a comparison between $R^2$ values for RF and GB models against other composition-based representation schemes from literature. We again see the GB model marginally outperform the RF model at both tasks. Comparison against other representations confirms the superior predictive performance of our selected features. Supplementary Fig. S11 shows parity plots comparing the oxidation and reduction potentials predicted using GB models against those calculated directly from DFT energies. We see excellent agreement between the two sets of values with no clear outliers.

**Table 5:** The cross-validated performance of our RF and GB models trained using 28 input features on the oxidation and reduction potential prediction tasks. Also compared are three other composition-based representations from literature (with the same GB model in each case).

Features + model	Reduction potential	Oxidation potential	Vector length
Current work + GB	0.95	0.92	28
Current work + RF	0.93	0.91	28
Roost^90^ + neural networks	0.92	0.92	64
Magpie element features^12^ + GB	0.89	0.84	132
Element fractions + GB	0.87	0.87	103

**Figure 4:**
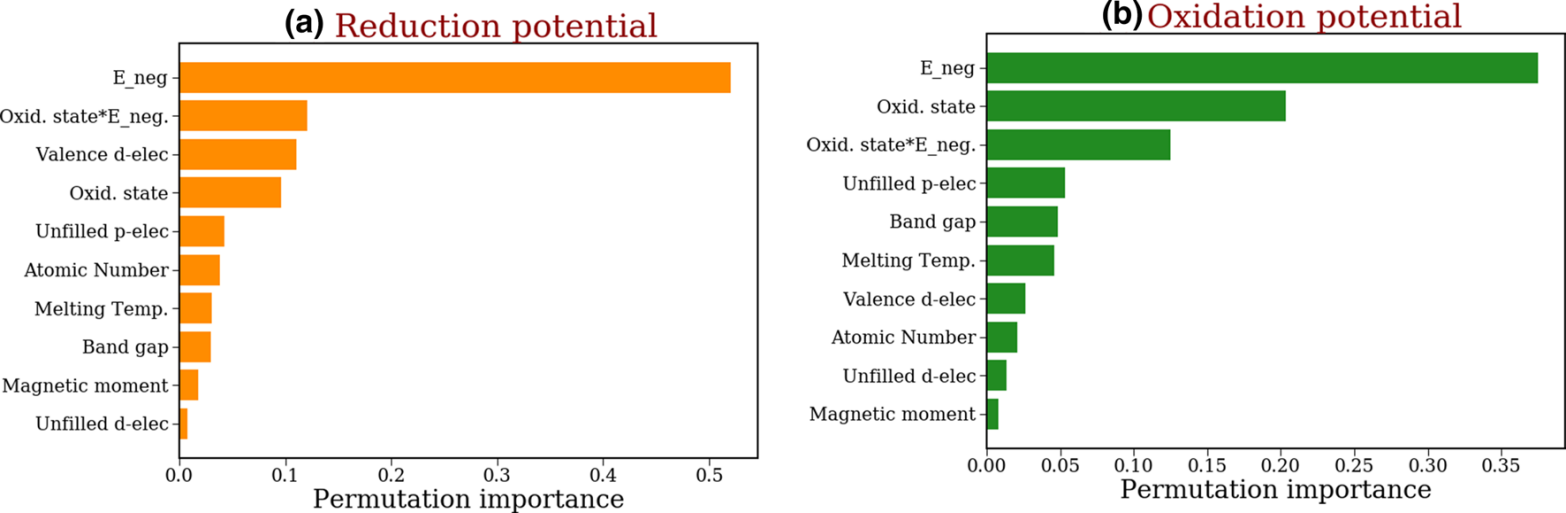
Permutation feature importance plots for the (**a**) reduction potential and (**b**) oxidation potential GB models. Features describing the same element property, measured through different statistics, are clustered together to avoid highly correlated features.

Shapley explanations for oxidation and reduction potential predictions are less intuitive because of the correlated nature of input features, which measure the same property through different statistics. Hence, we only perform PFI analysis to study overall feature importance for these GB models. We still need to cluster input features describing the same property into individual groups to understand the impact of each element property on the oxidation and reduction potential predictions. Additionally, we also combine the oxidation state features into two groups as shown in Fig. 4.

We see that *electronegativity* features have the largest impact on predictions of oxidation and reduction potentials. The electronegativity of an element represents its tendency to attract electrons, and largely determines whether it gets oxidized or reduced. Hence, the major influence on calculated oxidation and reduction potentials is expected. Additionally, the new oxidation state features introduced in this work also seem to be quite effective at predicting electrochemical windows. These features account for the variations in oxidation states of multivalent elements, making them excellent predictors of stability. Other input features that have a noticeable impact include the number of *valence d-electrons* and *unfilled p-electrons*.

It is important to point out that PFI and SHAP values are not intrinsic predictors of feature importance. They are model dependent, and only represent the importance a given value or feature has on predictions made using that particular model. However, for accurate models with physically motivated and interpretable features, these results can still be useful. For example, $\hbox {Li}_{{10}}\hbox {SiP}_2\hbox {O}_{{12}}$ (LSiPO) has a 3D barrier of 0.305 eV. We would expect a compound with the same crystal structure as LSiPO, but with larger sublattice ions, to exhibit a higher 3D barrier due to its large SPF, all other features being equal. We can test this by substituting Sn for Si in the structure. Sn belongs to the same group (IVA) and has a similar electronegativity as Si (1.96 *vs.* 1.90), but a bigger ionic radius. By simply changing the SPF, we find that the 3D barrier of LSnPO increases to 0.518 eV. Similarly, if we substitute the O in LSiPO with S instead, we would expect the new compound to have a lower ENS due to the lower electronegativity of S compared to O (2.58 *vs.* 3.44), as well as a higher SPF due to the larger S ions. This would also drive the barrier higher, which is exactly what we see for LSiPS, whose 3D barrier is 0.457 eV.

The above example shows that, even though PFI analysis and Shapley explanations do not necessarily provide causality, they can still serve as a useful guide for designing new compounds with tailored 3D barriers. Besides substitutions, the introduction of intrinsic defects and dopants can also be used to alter the geometry, electronegativity, and the Li fraction in the compounds. Such an approach has the potential to enable the discovery of promising new solid electrolytes and will be the focus of our future work.

## Summary

In summary, we developed a materials informatics approach to explore and identify promising candidates for solid electrolytes and protective anode coatings in all-solid-state Li batteries. By combining high-fidelity DFT calculations with quick BV-based computations, we generated a database of battery-related materials properties for all Li compounds on Materials Project. We screened through the database to identify over 250 electrolyte and 26 anode coating candidates that span a wide range of structures and compositions. We found new compounds like $\hbox {Li}_9\hbox {S}_3\hbox {N}$, $\hbox {LiAlB}_2\hbox {O}_5$, $\hbox {LiYO}_2$, $\hbox {LiSbF}_4$, and $\hbox {Sr}_4\hbox {Li}(\hbox {BN}_2)_3$, that have extremely low 3D barriers, and provide a good balance of electrochemical stability and fast ionic migration.

We also trained machine learning models, using ensemble methods, to predict 3D migration barriers and oxidation and reduction potentials for Li compounds, to identify promising compounds outside our database. By using an assorted set of physically motivated features, we showed that our models can achieve high accuracy combined with interpretability. We compared our carefully selected features against common descriptors from literature to show that our features achieved better performance. Additionally, we explained individual predictions and provided model-level insights, useful for designing new electrolyte and coating materials in the future.

Our database and the subsequent screening, machine learning, and model interpretation techniques, together, provide a comprehensive strategy to efficiently explore the Li compound space for promising solid electrolyte candidates. Our approach has the potential to accelerate the discovery and design of novel electrolyte and coating materials for Li ASSB. Future work will involve designing and testing new Li compounds using the trained models, as well as accurate DFT simulations and experiments to perform rigorous validation of the screening and machine learning results. We also plan to extend our database to include other properties such as elastic moduli, which are predictors of dendrite formation, and stability in air and water in the future.

## Supplementary Information

Supplementary Information.

## Data Availability

Data not found in the main text or SI is available from the corresponding authors upon request. The developed ML model was implemented using the scikit-learn library in python. All the scripts for generating input features for the ML models are also available upon request from the corresponding authors.

## References

[1] Pasta M, Armstrong D, Brown ZL, Bu J, Castell MR, Chen P, Cocks A, Corr SA, Cussen EJ, Darnbrough E (2020). 2020 roadmap on solid-state batteries. J. Phys..

[2] Manthiram A, Yu X, Wang S (2017). Lithium battery chemistries enabled by solid-state electrolytes. Nat. Rev. Mater..

[3] Hwang J-Y, Park S-J, Yoon CS, Sun Y-K (2019). Customizing a Li-metal battery that survives practical operating conditions for electric vehicle applications. Energy Environ. Sci..

[4] Du Z, Wood DL, Belharouak I (2019). Enabling fast charging of high energy density Li-ion cells with high lithium ion transport electrolytes. Electrochem. Commun..

[5] Wang X (2020). Toward high-energy-density lithium metal batteries: Opportunities and challenges for solid organic electrolytes. Adv. Mater..

[6] Radin MD, Hy S, Sina M, Fang C, Liu H, Vinckeviciute J, Zhang M, Whittingham MS, Meng YS, Van der Ven A (2017). Narrowing the gap between theoretical and practical capacities in Li-ion layered oxide cathode materials. Adv. Energy Mater..

[7] Folkson R (2014). Alternative Fuels and Advanced Vehicle Technologies for Improved Environmental Performance: Towards Zero Carbon Transportation.

[8] Jain A, Ong SP, Hautier G, Chen W, Richards WD, Dacek S, Cholia S, Gunter D, Skinner D, Ceder G (2013). Commentary: The Materials Project: A materials genome approach to accelerating materials innovation. Appl. Mater..

[9] Kirklin S, Saal JE, Meredig B, Thompson A, Doak JW, Aykol M, Rühl S, Wolverton C (2015). The Open Quantum Materials Database (OQMD): Assessing the accuracy of DFT formation energies. NPJ Comput. Mater..

[10] Curtarolo S, Setyawan W, Wang S, Xue J, Yang K, Taylor RH, Nelson LJ, Hart GL, Sanvito S, Buongiorno-Nardelli M (2012). AFLOWLIB. ORG: A distributed materials properties repository from high-throughput ab initio calculations. Comput. Mater. Sci..

[11] Ward L, Dunn A, Faghaninia A, Zimmermann NE, Bajaj S, Wang Q, Montoya J, Chen J, Bystrom K, Dylla M (2018). Matminer: An open source toolkit for materials data mining. Comput. Mater. Sci..

[12] Ward L, Agrawal A, Choudhary A, Wolverton C (2016). A general-purpose machine learning framework for predicting properties of inorganic materials. NPJ Comput. Mater..

[13] Ramprasad R, Batra R, Pilania G, Mannodi-Kanakkithodi A, Kim C (2017). Machine learning in materials informatics: Recent applications and prospects. NPJ Comput. Mater..

[14] Jain A, Hautier G, Ong SP, Persson K (2016). New opportunities for materials informatics: Resources and data mining techniques for uncovering hidden relationships. J. Mater. Res..

[15] Zhou Q, Tang P, Liu S, Pan J, Yan Q, Zhang S-C (2018). Learning atoms for materials discovery. Proc. Natl. Acad. Sci. USA.

[16] Xie T, Grossman JC (2018). Crystal graph convolutional neural networks for an accurate and interpretable prediction of material properties. Phys. Rev. Lett..

[17] Isayev O, Fourches D, Muratov EN, Oses C, Rasch K, Tropsha A, Curtarolo S (2015). Materials cartography: Representing and mining materials space using structural and electronic fingerprints. Chem. Mater..

[18] Ye W, Chen C, Dwaraknath S, Jain A, Ong SP, Persson KA (2018). Harnessing the Materials Project for machine-learning and accelerated discovery. MRS Bull..

[19] Meredig B, Agrawal A, Kirklin S, Saal JE, Doak JW, Thompson A, Zhang K, Choudhary A, Wolverton C (2014). Combinatorial screening for new materials in unconstrained composition space with machine learning. Phys. Rev. B.

[20] Javed SG, Khan A, Majid A, Mirza AM, Bashir J (2007). Lattice constant prediction of orthorhombic ABO 3 perovskites using support vector machines. Comput. Mater. Sci.

[21] Pilania G, Wang C, Jiang X, Rajasekaran S, Ramprasad R (2013). Accelerating materials property predictions using machine learning. Sci. Rep..

[22] Liu Y, Zhao T, Ju W, Shi S (2017). Materials discovery and design using machine learning. J. Materiom..

[23] Rupp M, Tkatchenko A, Müller K-R, von Lilienfeld OA (2012). Fast and accurate modeling of molecular atomization energies with machine learning. Phys. Rev. Lett..

[24] Huo, H., & Rupp, M. Unified representation for machine learning of molecules and crystals (2017). arXiv preprint arXiv:1704.06439.

[25] von Lilienfeld OA, Ramakrishnan R, Rupp M, Knoll A (2015). Fourier series of atomic radial distribution functions: A molecular fingerprint for machine learning models of quantum chemical properties. Int. J. Quantum Chem..

[26] Montavon, G., Hansen, K., Fazli, S., Rupp, M., Biegler, F., Ziehe, A., Tkatchenko, A., Lilienfeld, A. V., & Müller, K.- R. Learning invariant representations of molecules for atomization energy prediction. In *Advances in Neural Information Processing Systems* 440–448 (2012)

[27] Honrao S, Anthonio BE, Ramanathan R, Gabriel JJ, Hennig RG (2019). Machine learning of ab-initio energy landscapes for crystal structure predictions. Comput. Mater. Sci..

[28] Honrao SJ, Xie SR, Hennig RG (2020). Augmenting machine learning of energy landscapes with local structural information. J. Appl. Phys..

[29] Faber F, Lindmaa A, von Lilienfeld OA, Armiento R (2015). Crystal structure representations for machine learning models of formation energies. Int. J. Quantum Chem..

[30] Wu H, Lorenson A, Anderson B, Witteman L, Wu H, Meredig B, Morgan D (2017). Robust FCC solute diffusion predictions from ab-initio machine learning methods. Comput. Mater. Sci..

[31] Lee J, Seko A, Shitara K, Nakayama K, Tanaka I (2016). Prediction model of band gap for inorganic compounds by combination of density functional theory calculations and machine learning techniques. Phys. Rev. B.

[32] Pilania G, Mannodi-Kanakkithodi A, Uberuaga B, Ramprasad R, Gubernatis J, Lookman T (2016). Machine learning bandgaps of double perovskites. Sci. Rep..

[33] Abraham K (2015). Prospects and limits of energy storage in batteries. J. Phys. Chem. Lett..

[34] Armand, M., Endres, F., MacFarlane, D. R., Ohno, H., & Scrosati, B. Ionic-liquid materials for the electrochemical challenges of the future. Materials for sustainable energy: a collection of peer-reviewed research and review articles from Nature Publishing Group 129–137 (2011).

[35] Giffin GA (2016). Ionic liquid-based electrolytes for beyond lithium battery technologies. J. Mater. Chem. A.

[36] Jónsson, H., Mills, G., & Jacobsen, K. W. Nudged elastic band method for finding minimum energy paths of transitions (1998).

[37] Meng YS, Arroyo-de Dompablo ME (2009). First principles computational materials design for energy storage materials in lithium ion batteries. Energy Environ. Sci..

[38] Xiao R, Li H, Chen L (2015). High-throughput design and optimization of fast lithium ion conductors by the combination of bond-valence method and density functional theory. Sci. Rep..

[39] Anurova N, Blatov V, Ilyushin G, Blatova O, Ivanov-Schitz A, Dem’yanets L (2008). Migration maps of Li+ cations in oxygen-containing compounds. Solid State Ionics.

[40] Polyakov V (2001). Visualization of conduction channels and the dynamics of ion transport in superionic conductors. Phys. Solid State.

[41] Adams S, Swenson J (2002). Pathway models for fast ion conductors by combination of bond valence and reverse Monte Carlo methods. Solid State Ionics.

[42] Adams S (2006). Bond valence analysis of structure-property relationships in solid electrolytes. J. Power Sources.

[43] Gao J, Chu G, He M, Zhang S, Xiao R, Li H, Chen L (2014). Screening possible solid electrolytes by calculating the conduction pathways using Bond Valence method. Sci. China Phys. Mech. Astron..

[44] Xiao R, Li H, Chen L (2015). Candidate structures for inorganic lithium solid-state electrolytes identified by high-throughput bond-valence calculations. J. Materiom..

[45] Chen H, Wong LL, Adams S (2019). SoftBV: A software tool for screening the materials genome of inorganic fast ion conductors. Acta Crystallogr. B.

[46] Nestler T, Meutzner F, Kabanov AA, Zschornak M, Leisegang T, Meyer DC (2019). Combined theoretical approach for identifying battery materials: Al3+ mobility in oxides. Chem. Mater..

[47] Belsky A, Hellenbrandt M, Karen VL, Luksch P (2002). New developments in the Inorganic Crystal Structure Database (ICSD): Accessibility in support of materials research and design. Acta Crystallogr. B.

[48] He B, Ye A, Chi S, Mi P, Ran Y, Zhang L, Zou X, Pu B, Zhao Q, Zou Z (2020). CAVD, towards better characterization of void space for ionic transport analysis. Sci. Data.

[49] He B, Chi S, Ye A, Mi P, Zhang L, Pu B, Zou Z, Ran Y, Zhao Q, Wang D (2020). High-throughput screening platform for solid electrolytes combining hierarchical ion-transport prediction algorithms. Sci. Data.

[50] Zhang L, He B, Zhao Q, Zou Z, Chi S, Mi P, Ye A, Li Y, Wang D, Avdeev M (2020). A database of ionic transport characteristics for over 29,000 inorganic compounds. Adv. Funct. Mater..

[51] He X, Bai Q, Liu Y, Nolan AM, Ling C, Mo Y (2019). Crystal structural framework of lithium super-ionic conductors. Adv. Energy Mater..

[52] Richards WD, Miara LJ, Wang Y, Kim JC, Ceder G (2016). Interface stability in solid-state batteries. Chem. Mater..

[53] Zhu Y, He X, Mo Y (2015). Origin of outstanding stability in the lithium solid electrolyte materials: Insights from thermodynamic analyses based on first-principles calculations. ACS Appl. Mater. Interfaces.

[54] Xiao Y, Miara LJ, Wang Y, Ceder G (2019). Computational screening of cathode coatings for solid-state batteries. Joule.

[55] Aykol M, Kim S, Hegde VI, Snydacker D, Lu Z, Hao S, Kirklin S, Morgan D, Wolverton C (2016). High-throughput computational design of cathode coatings for Li-ion batteries. Nat. Commun..

[56] Nolan AM, Liu Y, Mo Y (2019). Solid-state chemistries stable with high-energy cathodes for lithium-ion batteries. ACS Energy Lett..

[57] Zhu Y, He X, Mo Y (2017). Strategies based on nitride materials chemistry to stabilize Li metal anode. Adv. Sci..

[58] Tian Y (2020). Promises and challenges of next-generation Beyond Li-ion batteries for electric vehicles and grid decarbonization. Chem. Rev..

[59] Sendek AD, Yang Q, Cubuk ED, Duerloo K-AN, Cui Y, Reed EJ (2017). Holistic computational structure screening of more than 12000 candidates for solid lithium-ion conductor materials. Energy Environ. Sci..

[60] Sendek AD, Cubuk ED, Antoniuk ER, Cheon G, Cui Y, Reed EJ (2018). Machine learning-assisted discovery of solid Li-ion conducting materials. Chem. Mater..

[61] Thangadurai V, Narayanan S, Pinzaru D (2014). Garnet-type solid-state fast Li ion conductors for Li batteries: Critical review. Chem. Soc. Rev..

[62] Adams S (2001). Relationship between bond valence and bond softness of alkali halides and chalcogenides. Acta Crystallogr. B.

[63] Chen H, Adams S (2017). Bond softness sensitive bond-valence parameters for crystal structure plausibility tests. IUCrJ.

[64] Ceder G, Ong SP, Wang Y (2018). Predictive modeling and design rules for solid electrolytes. Mrs Bull..

[65] Nishitani Y, Adams S, Ichikawa K, Tsujita T (2018). Evaluation of magnesium ion migration in inorganic oxides by the bond valence site energy method. Solid State Ionics.

[66] Ong SP, Wang L, Kang B, Ceder G (2008). Li–Fe–P–O2 phase diagram from first principles calculations. Chem. Mater..

[67] Aydinol M, Kohan A, Ceder G, Cho K, Joannopoulos J (1997). Ab initio study of lithium intercalation in metal oxides and metal dichalcogenides. Phys. Rev. B.

[68] Chan M, Ceder G (2010). Efficient band gap prediction for solids. Phys. Rev. Lett..

[69] Liu Z, Fu W, Payzant EA, Yu X, Wu Z, Dudney NJ, Kiggans J, Hong K, Rondinone AJ, Liang C (2013). Anomalous high ionic conductivity of nanoporous $$\beta $$-Li3PS4. J. Am. Chem. Soc..

[70] Seino Y, Ota T, Takada K, Hayashi A, Tatsumisago M (2014). A sulphide lithium super ion conductor is superior to liquid ion conductors for use in rechargeable batteries. Energy Environ. Sci..

[71] Whiteley JM, Woo JH, Hu E, Nam K-W, Lee S-H (2014). Empowering the lithium metal battery through a silicon-based superionic conductor. J. Electrochem. Soc..

[72] Boulineau S, Courty M, Tarascon J-M, Viallet V (2012). Mechanochemical synthesis of Li-argyrodite Li6PS5X (X= Cl, Br, I) as sulfur-based solid electrolytes for all solid state batteries application. Solid State Ionics.

[73] Iddir H, Curtiss LA (2010). Li ion diffusion mechanisms in bulk monoclinic Li2CO3 crystals from density functional studies. J. Phys. Chem. C.

[74] Shi S, Qi Y, Li H, Hector LG (2013). Defect thermodynamics and diffusion mechanisms in Li2CO3 and implications for the solid electrolyte interphase in Li-ion batteries. J. Phys. Chem. C.

[75] Guo R, Gallant BM (2020). Li2O solid electrolyte interphase: Probing transport properties at the chemical potential of lithium. Chem. Mater..

[76] Liu F, Wang L, Zhang Z, Shi P, Feng Y, Yao Y, Ye S, Wang H, Wu X, Yu Y (2020). A mixed lithium-ion conductive Li2S/Li2Se protection layer for stable lithium metal anode. Adv. Funct. Mater..

[77] Zou Y, Petric A (1993). Structure and conductivity of zirconium-doped polycrystalline lithium yttrium oxide. Mater. Res. Bull..

[78] Zaiß T, Ortner M, Murugan R, Weppner W (2010). Fast ionic conduction in cubic hafnium garnet Li 7 La 3 Hf 2 O 12. Ionics.

[79] Breiman L (2001). Random forests. Mach. Learn..

[80] Friedman JH (2001). Greedy function approximation: A gradient boosting machine. Ann. Stat..

[81] Friedman JH (2002). Stochastic gradient boosting. Comput. Stat. Data Anal..

[82] Pedregosa F, Varoquaux G, Gramfort A, Michel V, Thirion B, Grisel O, Blondel M, Prettenhofer P, Weiss R, Dubourg V (2011). Scikit-learn: Machine learning in Python. J. Mach. Learn. Res..

[83] Ward L, Liu R, Krishna A, Hegde VI, Agrawal A, Choudhary A, Wolverton C (2017). Including crystal structure attributes in machine learning models of formation energies via Voronoi tessellations. Phys. Rev. B.

[84] Willems TF, Rycroft CH, Kazi M, Meza JC, Haranczyk M (2012). Algorithms and tools for high-throughput geometry-based analysis of crystalline porous materials. Microporous Mesoporous Mater..

[85] Lundberg, S., Lee, S. -I. A unified approach to interpreting model predictions (2017). arXiv preprint arXiv:1705.07874.

[86] Parr, T. P., & Turgutlu, K. Feature importances for scikit-learn machine learning models, https://github.com/parrt/random-forest-importances.

[87] Morgan D, Van der Ven A, Ceder G (2003). Li conductivity in Li x MPO 4 (M= Mn, Fe Co, Ni) olivine materials. Electrochem. Solid State Let..

[88] Ouyang C, Shi S, Wang Z, Huang X, Chen L (2004). First-principles study of Li ion diffusion in LiFePO 4. Phys. Rev. B.

[89] Lundberg SM, Nair B, Vavilala MS, Horibe M, Eisses MJ, Adams T, Liston DE, Low DK-W, Newman S-F, Kim J (2018). Explainable machine-learning predictions for the prevention of hypoxaemia during surgery. Nat. Biomed. Eng..

[90] Goodall, R. E., & Lee, A. A. Predicting materials properties without crystal structure: Deep representation learning from stoichiometry (2019). arXiv preprint arXiv:1910.00617. 10.1038/s41467-020-19964-7 PMC7722901 33293567

