# Supplementary material for: Discovery of novel Li SSE and anode coatings using interpretable machine learning and high-throughput multi-property screening
Source: Sci Rep. 2021 Aug 13;11:16484. doi: 10.1038/s41598-021-94275-5 (PMC8363752; doi:10.1038/s41598-021-94275-5)
Supplement: Supplementary file 1 — Supplementary Information. [file 41598_2021_94275_MOESM1_ESM.pdf]

Supplementary Information: Discovery of Novel Li SSE and Anode Coatings Using Interpretable Machine Learning and High-Throughput Multi-Property Screening

Shreyas J. Honrao,[1] Xin Yang,[2] Balachandran Radhakrishnan,[1] Shigemasa Kuwata,[2] Hideyuki Komatsu,[3] Atsushi Ohma,[3] Maarten Sierhuis,[2] and John W. Lawson[4]

[1]*KBR Wyle, Intelligent Systems Division, NASA Ames Research Center, Moffett Field, CA 94035, USA*

[2]*Research Division, Nissan North America, Santa Clara, CA 95051, USA*

[3]*Research Division, Nissan Motor Company, Yokosuka, Kanagawa 237-8523, Japan*

[4]*Intelligent Systems Division, NASA Ames Research Center, Moffett Field, CA 94035, USA*

Email: grbala@umich.edu, john.w.lawson@nasa.gov

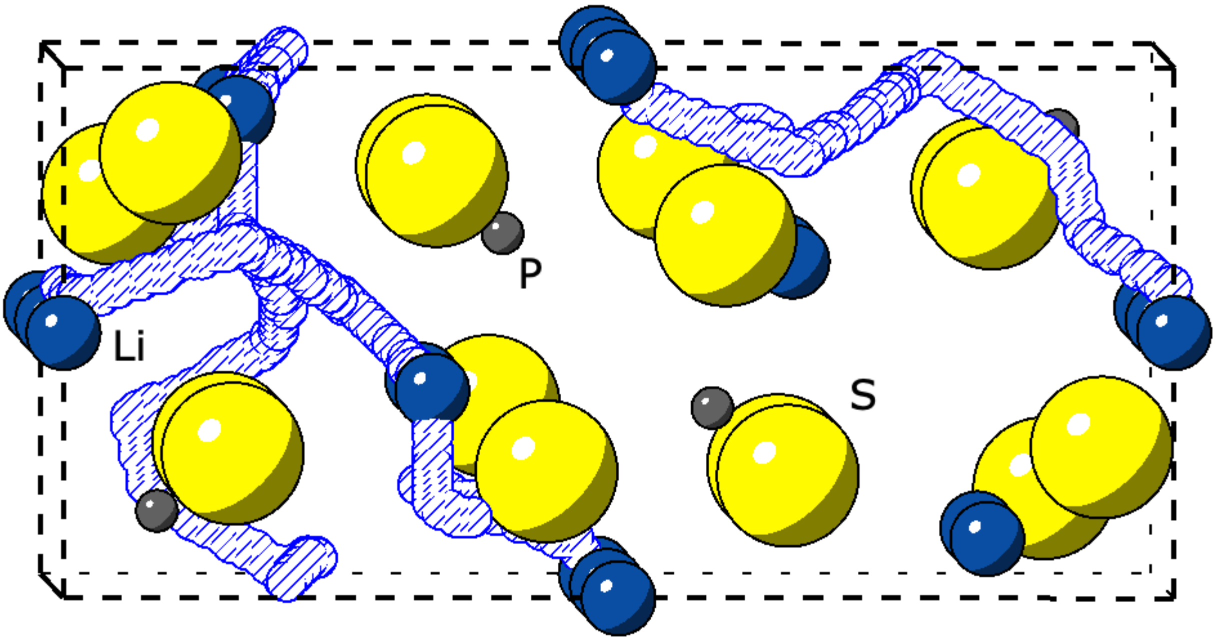

FIG. S1: Illustration of the 3D pathway for Li^+ migration in Li_3PS_4, computed using the softBV tool. The pathway represents the lowest energy isosurface that percolates through all three directions of the unit cell.

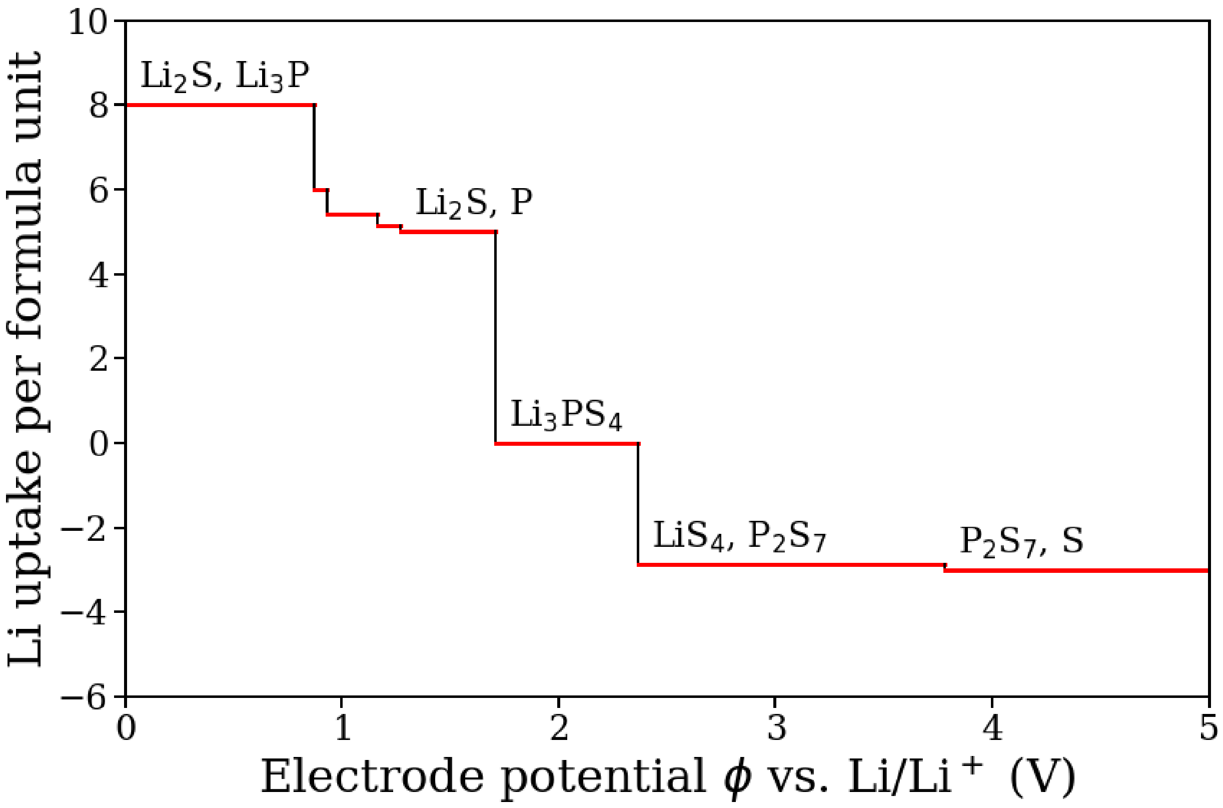

FIG. S2: Voltage profile depicting the lithiation and delithiation of Li_3PS_4. The electrochemical stability window of Li_3PS_4 extends from 1.7-2.2 V, the voltage range corresponding to zero Li uptake. Text indicates the predicted phase equilibria across various voltage ranges.

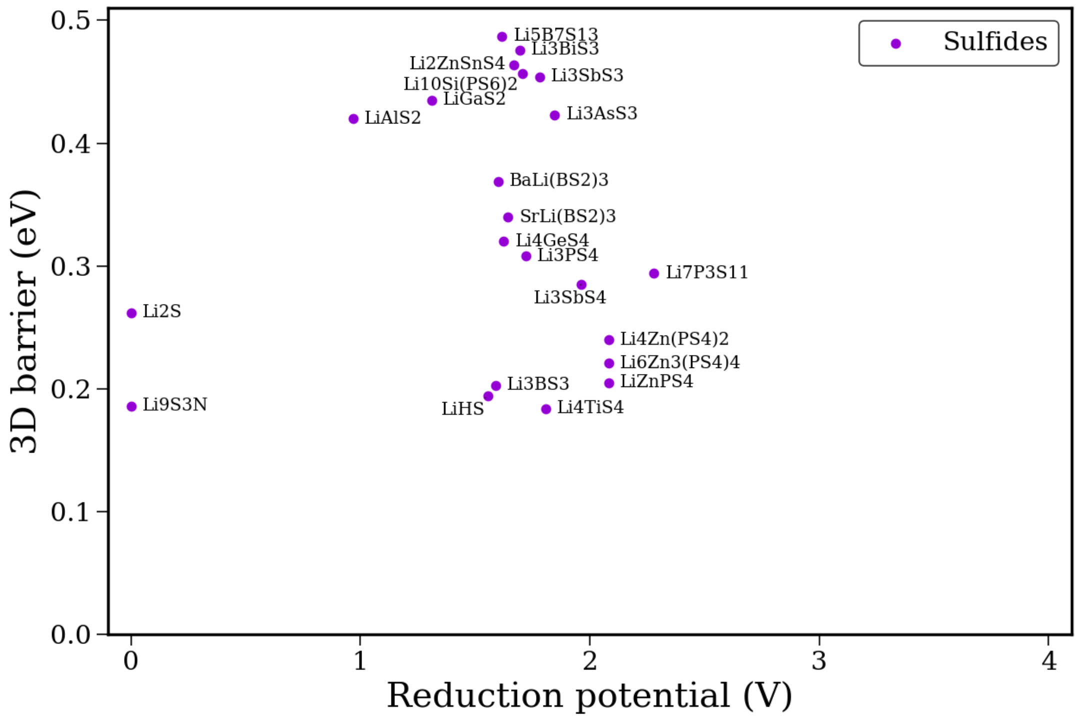

FIG. S3: Sulfide compounds identified as promising solid electrolyte candidates by our screening algorithm. Apart from Li_2S and Li_9S_3N, all other sulfides have high reduction potentials and are thus unstable against Li metal. [3D barrier $\leq$ 0.5 eV, $E_{hull} \leq$ 30 meV, $E_g \geq$ 1 eV]

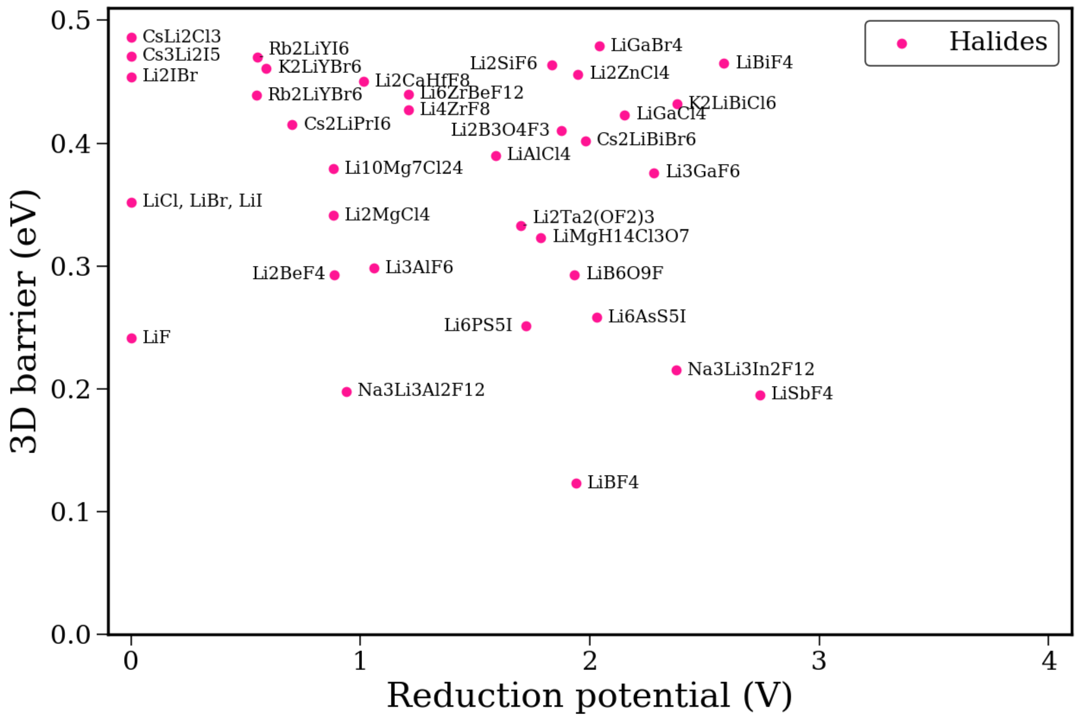

FIG. S4: Halide compounds identified as promising solid electrolyte candidates by our screening algorithm. $LiBF_4$ and $LiSbF_4$ are predicted to have the lowest 3D barriers. [3D barrier $\leq$ 0.5 eV, $E_{hull} \leq 30$ meV, $E_g \geq 1$ eV]

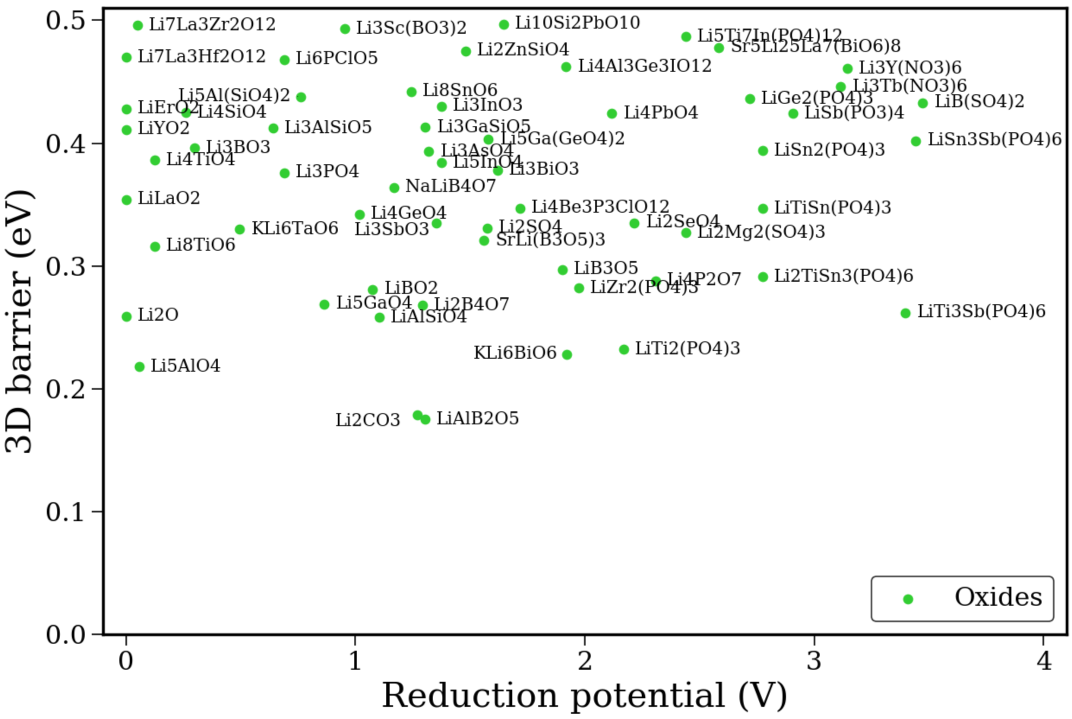

FIG. S5: Oxide compounds identified as promising solid electrolyte candidates by our screening algorithm. Several oxides also have low reduction potentials, and can act as good anode coatings. [3D barrier ≤ 0.5 eV, $E_{hull} \leq 30$ meV, $E_g \geq 1$ eV]

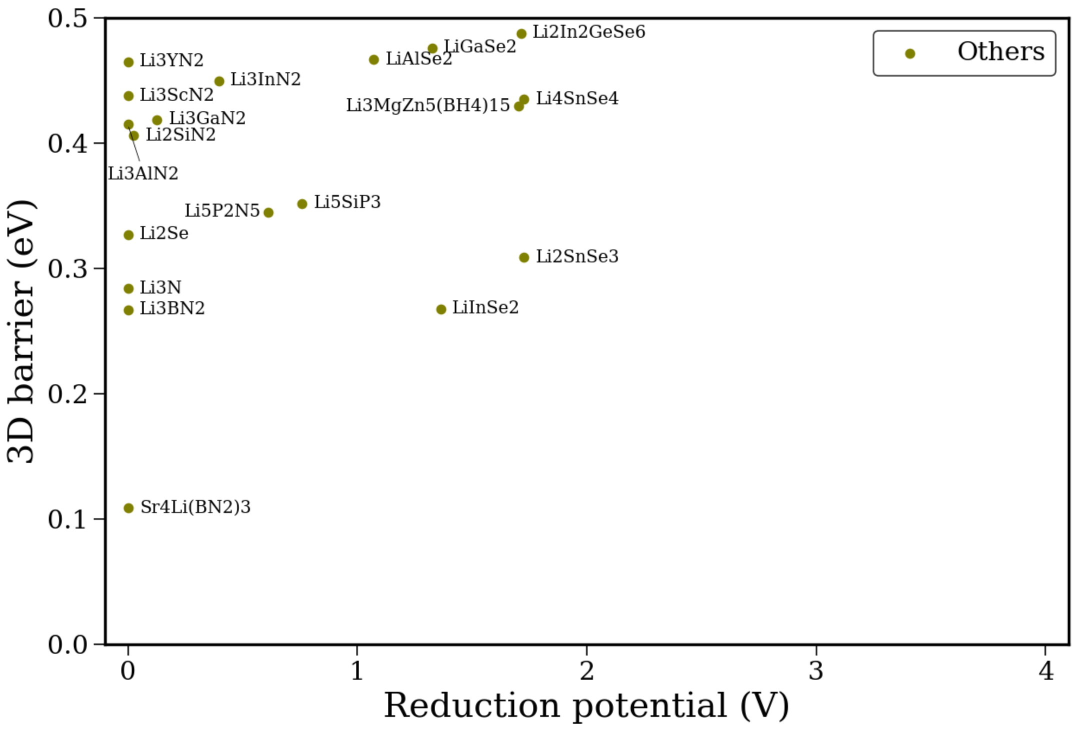

FIG. S6: Other compounds identified as promising solid electrolyte candidates by our screening algorithm. Nitrides have the lowest reduction potentials among other compounds. [3D barrier ≤ 0.5 eV, $E_{hull} \leq 30$ meV, $E_g \geq 1$ eV]

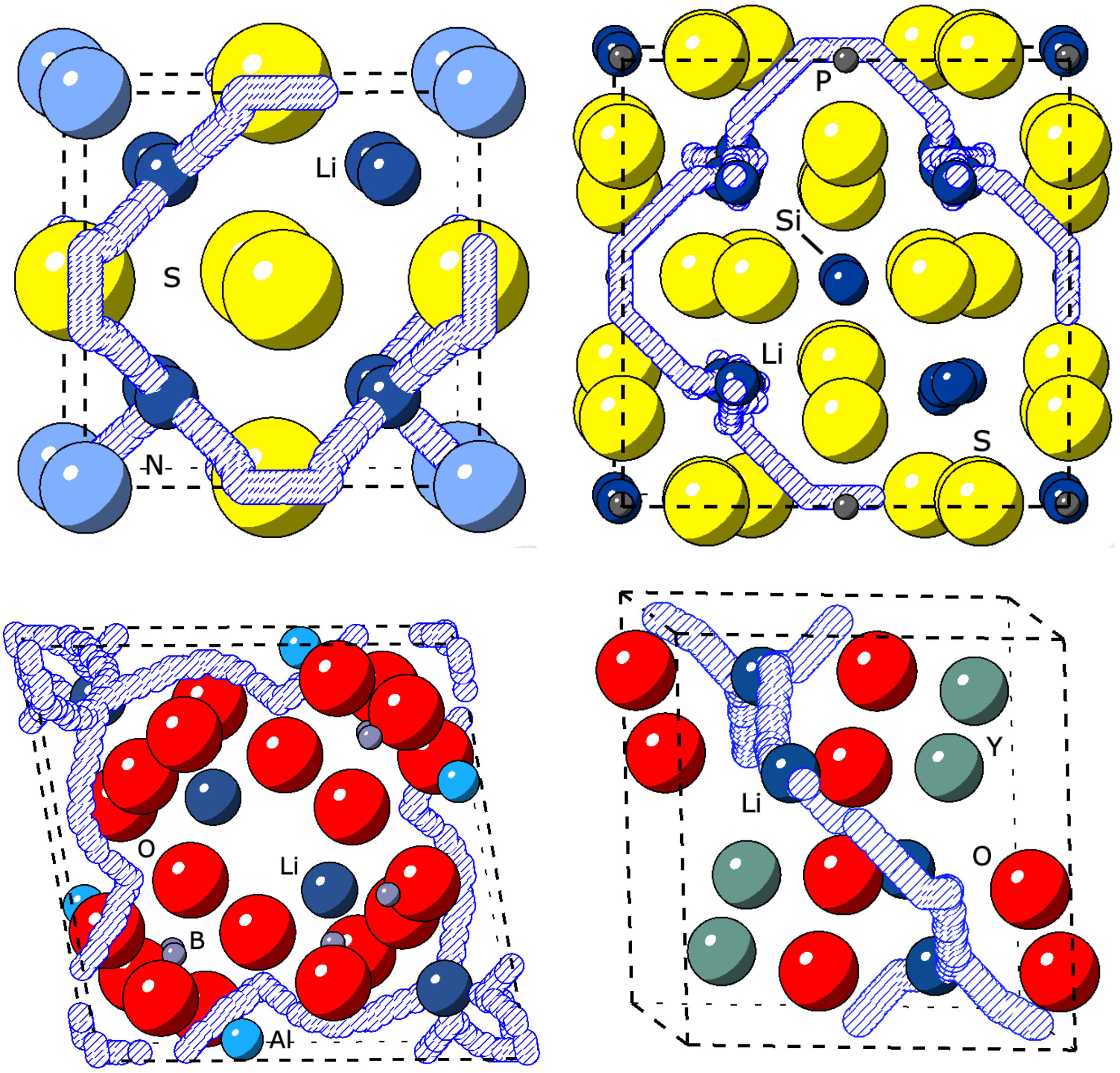

FIG. S7: Illustration of the 3D pathway for Li^+ migration in a few promising electrolyte and anode coating candidates identified through our screening approach: (a) Li_9S_3N, (b) $Li_{10}SiP_2S_{12}$, (c) $LiAlB_2O_5$, and (d) $LiYO_2$.

TABLE S1: Compounds identified as promising candidates for coatings on Li metal anode, arranged in order of increasing 3D barriers. [Reduction potential (*vs.* Li) = 0 V, electrochemical window ≥ 1 V, 3D barrier ≤ 1 eV, $E_{hull} \leq 30$ meV, $E_g \geq 1$ eV]

No.	mp_id	Formula	3D barrier (eV)	electochemical window (V)
1.	mp-9723	$Sr4Li(BN_2)_3$	0.109	1.39
2.	mp-1960	Li_2O	0.259	2.90
3.	mp-1153	Li_2S	0.262	2.13
4.	mp-2286	Li_2Se	0.327	1.89
5.	mp-1185319	LiCl	0.349	4.25
6.	mp-976280	LiBr	0.350	3.14
7.	mp-570935	LiI	0.352	2.47
8.	mp-756544	$LiLaO_2$	0.354	2.90
9.	mp-7020	$LiYO_2$	0.411	2.92
10.	mp-752922	Li_8HfO_6	0.419	2.90
11.	mp-10970	$LiErO_2$	0.428	3.00
12.	mp-1222669	Li_2IBr	0.454	2.47
13.	mp-1198622	$Li_7La_3Hf_2O_{12}$	0.470	3.02
14.	mp-1181918	$Cs_3Li_2I_5$	0.471	2.35
15.	mp-1190687	$CsLi_2Cl_3$	0.486	4.25
16.	mp-1185301	LiF	0.536	6.36
17.	mp-1080534	$Cs_2Li_3I_5$	0.544	2.35
18.	mp-28237	$RbLiBr_2$	0.590	3.14
19.	mp-1190087	$CsLiI_2$	0.601	2.35
20.	mp-1184020	$CsLi_3I_4$	0.668	2.35
21.	mp-28243	$RbLiCl_2$	0.703	4.25
22.	mp-606680	$CsLi_2Br_3$	0.774	2.96
23.	mp-569055	$CsLi_2I_3$	0.817	2.35
24.	mp-770805	$Li_6Hf_2O_7$	0.845	3.22
25.	mp-23057	$CsLiBr_2$	0.882	2.96
26.	mp-9610	Li_2CN_2	0.982	2.12

S1. MACHINE LEARNING FEATURES FOR PREDICTING 3D BARRIERS

A description of the 22 input features used in this work for predicting 3D barriers is given below.

S1.1. Li atomic fraction, Li

Ratio of number of lithium atoms (N_{Li}) to the total number of atoms (N_{atoms}) in the unit cell.

$$\text{Li} = \frac{N_{Li}}{N_{atoms}}$$

S1.2. Mean Li neighbor count, LNC [1]

Average number of neighbors within a radius of 4 Å for lithium atoms in the unit cell.

$$\text{LNC} = \frac{1}{N_{Li}} \sum_{j \neq i}^{N_{atoms}} \sum_{i \in \{Li\}}^{N_{Li}} 1\{r_{ij} < 4\text{Å}\}$$

S1.3. Mean Li-Li bonds per Li, LLB [1]

Average number of lithium neighbors within a radius of 4 Å for lithium atoms in the unit cell.

$$\text{LLB} = \frac{1}{N_{Li}} \sum_{j \in \{Li\}, j \neq i}^{N_{Li}} \sum_{i \in \{Li\}}^{N_{Li}} 1\{r_{ij} < 4\text{Å}\}$$

S1.4. Mean sublattice neighbor count, SNC [1]

Average number of neighbors within a radius of 4 Å for non-lithium atoms in the unit cell.

$$\text{SNC} = \frac{1}{N_{atoms} - N_{Li}} \sum_{j \neq i}^{N_{atoms}} \sum_{i \notin \{Li\}}^{N_{atoms} - N_{Li}} 1\{r_{ij} < 4\text{Å}\}$$

S1.5. Mean sublattice bond ionicity, SBI [1]

Average difference in the Pauling electronegativity of bonds (EN) between a sublattice atom and another atom within 4 Å of each other.

$$\text{SBI} = \frac{1}{N_{ij}} \sum_{j \neq i}^{N_{atoms}} \sum_{i \notin \{Li\}}^{N_{atoms}-N_{Li}} |\text{EN}_i - \text{EN}_j| \; 1\{r_{ij} < 4\text{Å}\}$$

S1.6. Mean Electronegativity of sublattice, ENS [1]

Average Pauling electronegativity (EN) of all sublattice atoms in the unit cell.

$$\text{ENS} = \frac{1}{N_{atoms} - N_{Li}} \sum_{i \notin \{Li\}}^{N_{atoms}-N_{Li}} \text{EN}_i$$

S1.7. Mean Li-Li separation distance, LLSD [1]

Average min. Li-Li distance for all lithium atoms in the unit cell.

$$\text{LLSD} = \frac{1}{N_{Li}} \sum_{i \in \{Li\}}^{N_{Li}} min_{j \in \{Li\}, j \neq i} \; \{r_{ij}\}$$

S1.8. Mean Li-anion separation distance, LASD [1]

Average min. Li-anion distance for all lithium atoms in the unit cell, where anion (A) refers to the atom with the highest electronegativity in the unit cell.

$$\text{LASD} = \frac{1}{N_{Li}} \sum_{i \in \{Li\}}^{N_{Li}} min_{j \in \{A\}} \; \{r_{ij}\}$$

S1.9. Mean anion-anion separation distance, AASD [1]

Average min. anion-anion distance for all anions in the unit cell, where anion (A) refers to the atom with the highest electronegativity in the unit cell.

$$\text{AASD} = \frac{1}{N_A} \sum_{i \in \{A\}}^{N_A} min_{j \in \{A\}, j \neq i} \; \{r_{ij}\}$$

S1.10. Mean neighbor distance variation, NDV [2]

Average standard deviation in bond lengths for all neighbors of a particular atom, weighted by the corresponding face area of the Voronoi cell. *Refer to the SI accompanying ref. [2] for details.*

S1.11. Mean ordering parameter shell 1, OP_1 [2]

Warren-Cowley-like ordering parameters [3] of the first neighbor shell, weighted by the corresponding face areas of the Voronoi cell for each neighboring atom in that shell. *Refer to the SI accompanying ref. [2] for details.*

S1.12. Mean ordering parameter shell 2, OP_2 [2]

Warren-Cowley-like ordering parameters [3] of the second neighbor shell, weighted by the corresponding face areas of the Voronoi cell for each neighboring atom in that shell. *Refer to the SI accompanying ref. [2] for details.*

S1.13. Mean ordering parameter shell 3, OP_3 [2]

Warren-Cowley-like ordering parameters [3] of the third neighbor shell, weighted by the corresponding face areas of the Voronoi cell for each neighboring atom in that shell. *Refer to the SI accompanying ref. [2] for details.*

S1.14. Diameter of largest free sphere, DLFS

The max. diameter of a sphere that can percolate across any one direction of the unit cell without intersecting any atoms. We calculate DLFS using the Zeo++ tool [4].

S1.15. Mean Straight Line Path Width, SLPW [1]

Average max. radius of the cylinder that connects each lithium atom with its nearest lithium neighbor without intersecting any other atoms.

S1.16. Sublattice packing fraction, SPF [1]

Ratio of total volume occupied by all sublattice atoms (V_i) to the total volume of the unit cell (V_{cell}).

$$\text{SPF} = \frac{1}{V_{cell}} \sum_{i \notin \{Li\}}^{N_{atoms}-N_{Li}} V_i$$

S1.17. Max packing efficiency, MPE [2]

The ratio of sum of volumes of largest spheres that fit inside the Voronoi cell around each atom (VS_i) to the total volume of the unit cell (V_{cell}).

$$\text{MPE} = \frac{1}{V_{cell}} \sum_{i \in \{atoms\}}^{N_{atoms}} \text{VS}_i$$

S1.18. XRD principal component 1, XRD_1

First principal component of the powder X-ray diffraction (XRD) pattern computed using pymatgen [5].

S1.19. XRD principal component 2, XRD_2

Second principal component of the powder X-ray diffraction (XRD) pattern computed using pymatgen [5].

S1.20. XRD principal component 3, XRD_3

Third principal component of the powder X-ray diffraction (XRD) pattern computed using pymatgen [5].

S1.21. XRD principal component 4, XRD_4

Fourth principal component of the powder X-ray diffraction (XRD) pattern computed using pymatgen [5].

S1.22. XRD principal component 5, XRD_5

Fifth principal component of the powder X-ray diffraction (XRD) pattern computed using pymatgen [5].

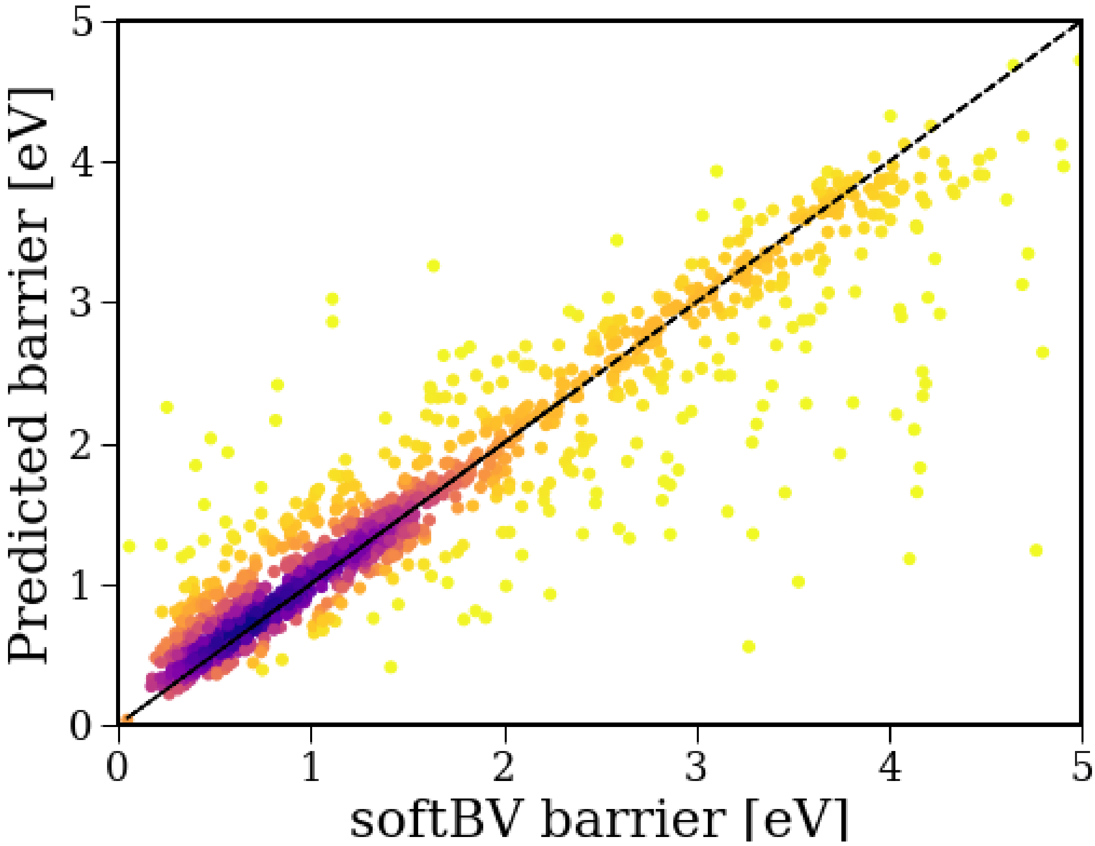

FIG. S8: Parity plot comparing the 3D barriers predicted using the GB model against barriers computed with softBV on the held-out test set. Good agreement can be seen between the two in the $0-1$ eV range, most relevant for practical applications.

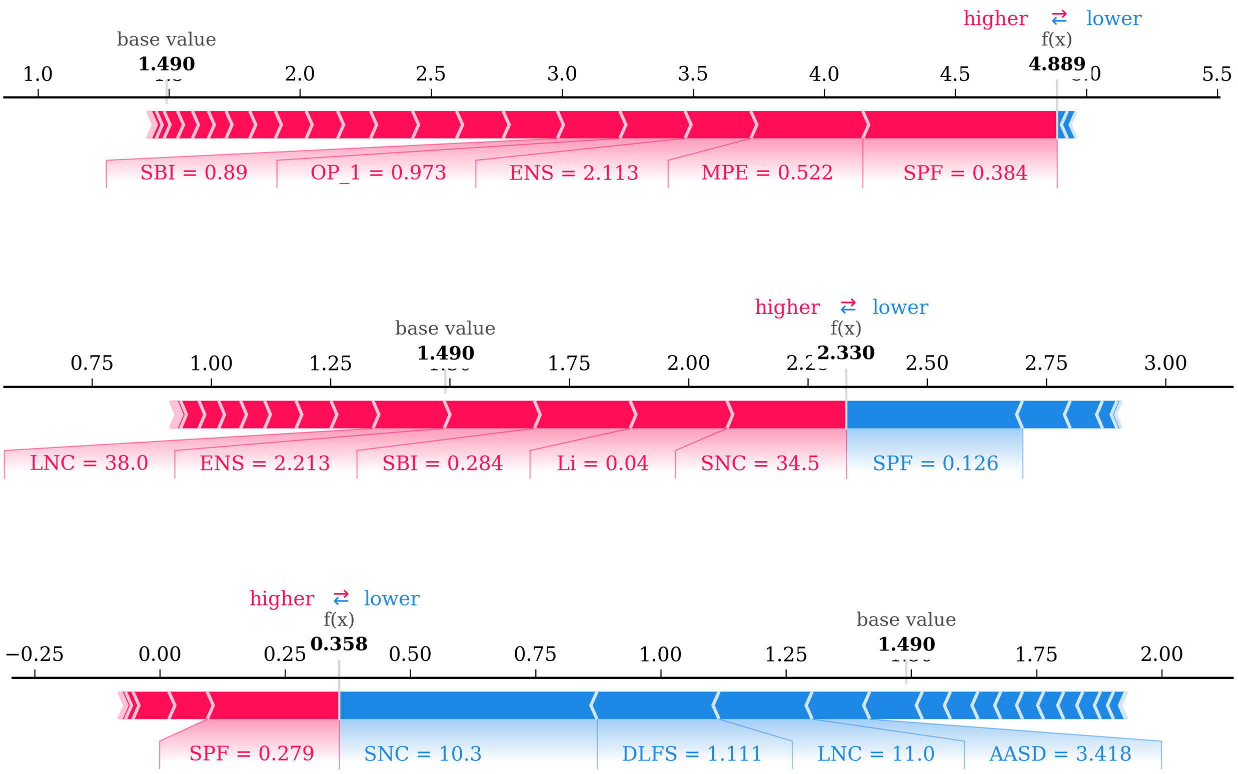

FIG. S9: Shapley explanations for individual predictions made by the GB model trained on 3D barriers. From top to bottom: $LiErSe_2$, $LiMgB_3(H_9N)_2$, and $SrLi(BS_2)_3$.

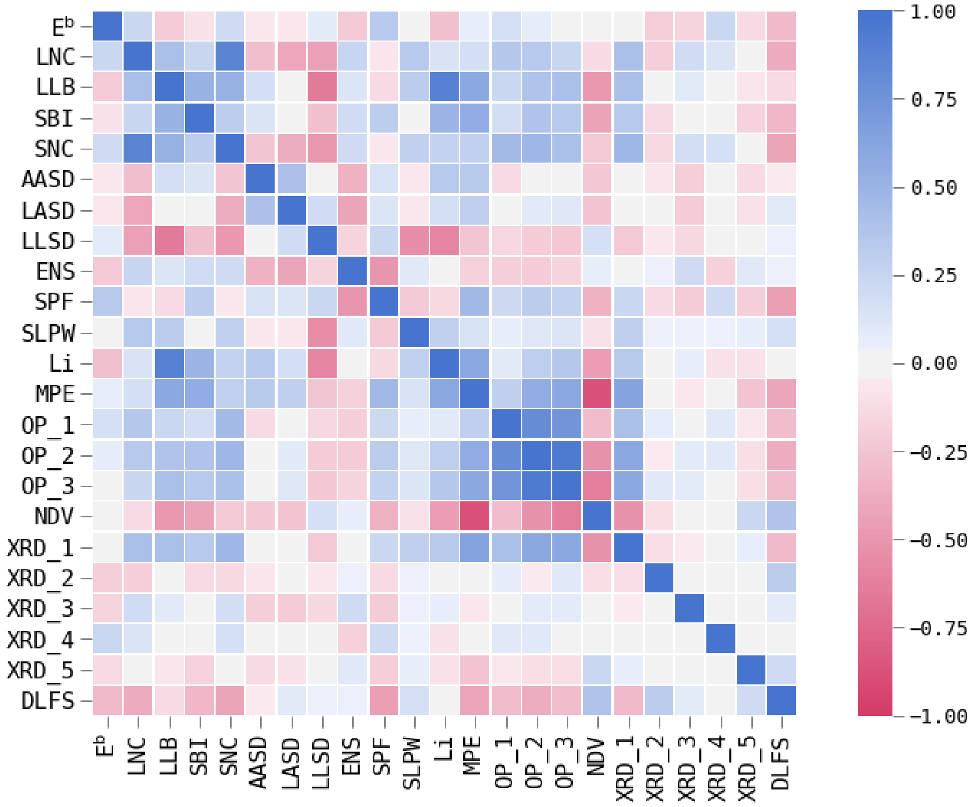

FIG. S10: Heatmap plot showing the Pearson correlation coefficients between each pair of input features and between individual features and the softBV 3D barrier (E^b). Blue and red boxes indicate positive and negative correlation, whereas darker and lighter shades represent strong and weak correlation.

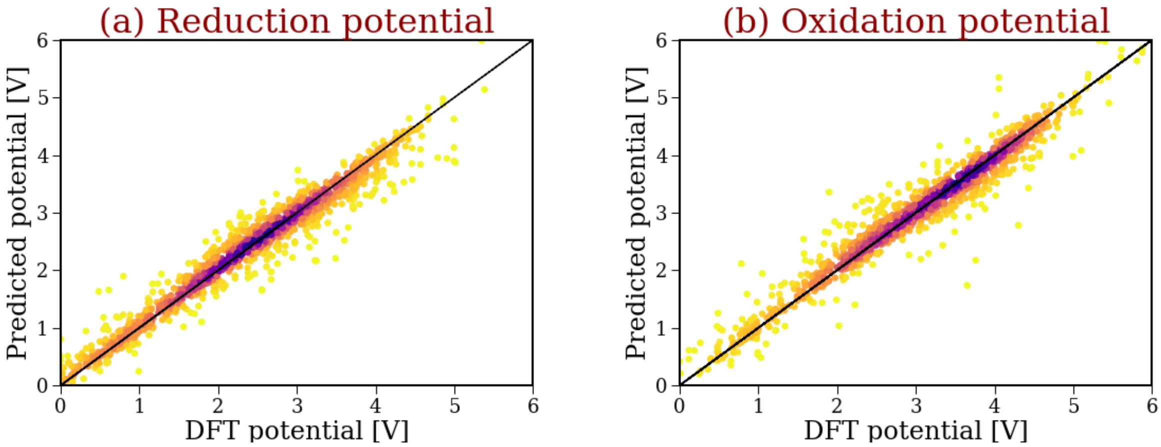

FIG. S11: Parity plots comparing the (a) reduction potentials and (b) oxidation potentials (*vs.* Li) predicted using the GB models against DFT values on the held-out test set.

[1] A. D. Sendek, Q. Yang, E. D. Cubuk, K.-A. N. Duerloo, Y. Cui, and E. J. Reed, Energy & Environmental Science **10**, 306 (2017).

[2] L. Ward, R. Liu, A. Krishna, V. I. Hegde, A. Agrawal, A. Choudhary, and C. Wolverton, Physical Review B **96**, 024104 (2017).

[3] J. Cowley, Physical Review **77**, 669 (1950).

[4] T. F. Willems, C. H. Rycroft, M. Kazi, J. C. Meza, and M. Haranczyk, Microporous and Mesoporous Materials **149**, 134 (2012).

[5] S. P. Ong, W. D. Richards, A. Jain, G. Hautier, M. Kocher, S. Cholia, D. Gunter, V. L. Chevrier, K. A. Persson, and G. Ceder, Computational Materials Science **68**, 314 (2013).
